# Supplementary material for: L-selectin Is Essential for Delivery of Activated CD8+ T Cells to Virus-Infected Organs for Protective Immunity
Source: Cell Rep. 2016 Jan 21;14(4):760–71. doi: 10.1016/j.celrep.2015.12.090 (PMC4742564; doi:10.1016/j.celrep.2015.12.090)
Supplement: Document S1. Supplemental Experimental Procedures, Figures S1 and S2, and Table S1 [file mmc1.pdf]

Cell Reports

Supplemental Information

L-selectin Is Essential for Delivery of Activated CD8$^+$ T Cells to Virus-Infected Organs for Protective Immunity

Rebar N. Mohammed, H. Angharad Watson, Miriam Vigar, Julia Ohme, Amanda Thomson, Ian R. Humphreys, and Ann Ager

Supplemental Information

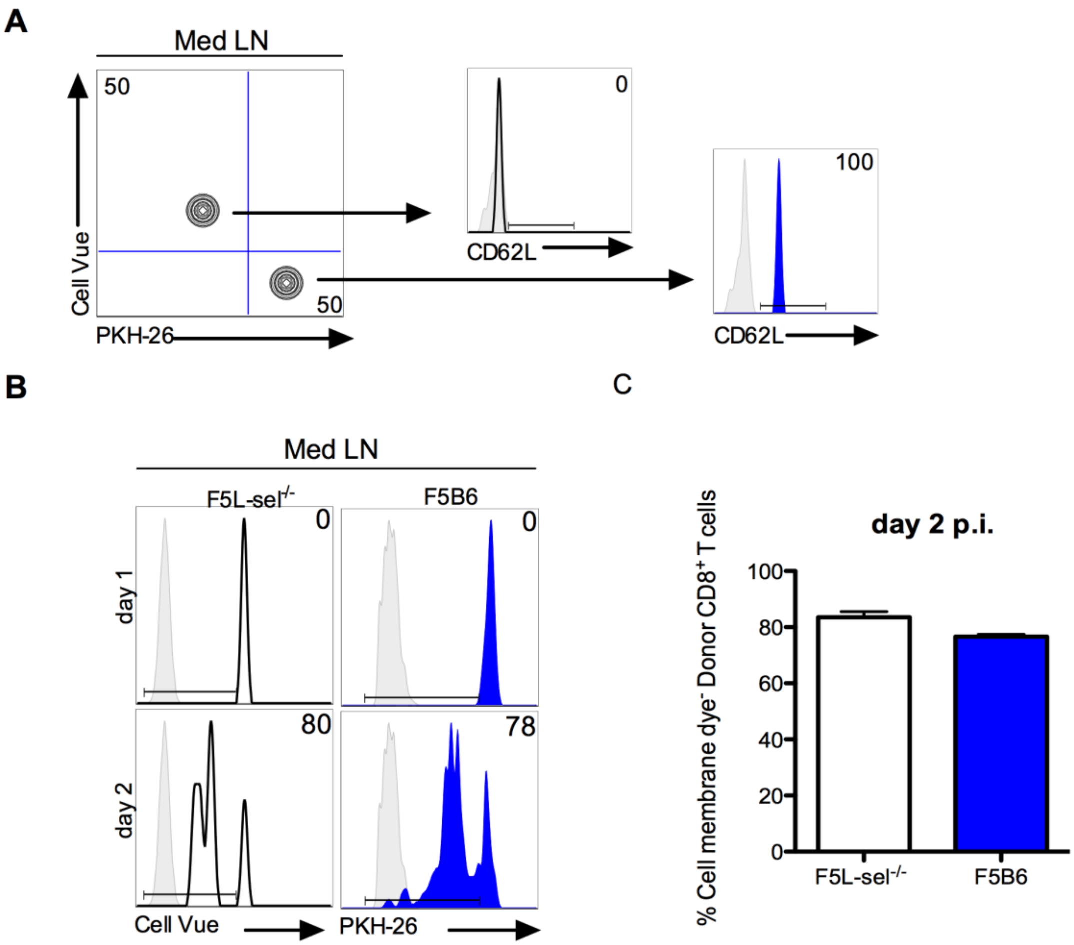

Figure S1, Related to Figure 2: L-selectin expression does not affect localization or priming of $CD8^+$ T cells in the mediastinal LN. Naïve F5L-$sel^{-/-}$ and F5B6 $CD8^+$ T cells were purified and stained with 2μM Cell Vue Claret and PKH-26 respectively. Cells were mixed 1:1 and total of 4×10^6 were transferred i.v. into CD90.1 B6 mice. Mice were infected with 2×10^6 pfu vaccNP or left un-infected. **(A)** Representative plot shows the percentage of F5B6 (PKH-26^+) and F5L-$sel^{-/-}$ (Cell Vue^+) $CD8^+$ T cells in the Mediastinal LN in un-infected mice 24h post transfer; the donor cells were confirmed by CD62L expression. **(B)** Representative histograms show the percentage cell tracker dye dilution at days 1 and 2 p.i. compared to the unstained cell, grey histograms. **(C)** Bar chart shows the mean + SEM dye dilution (n=3).

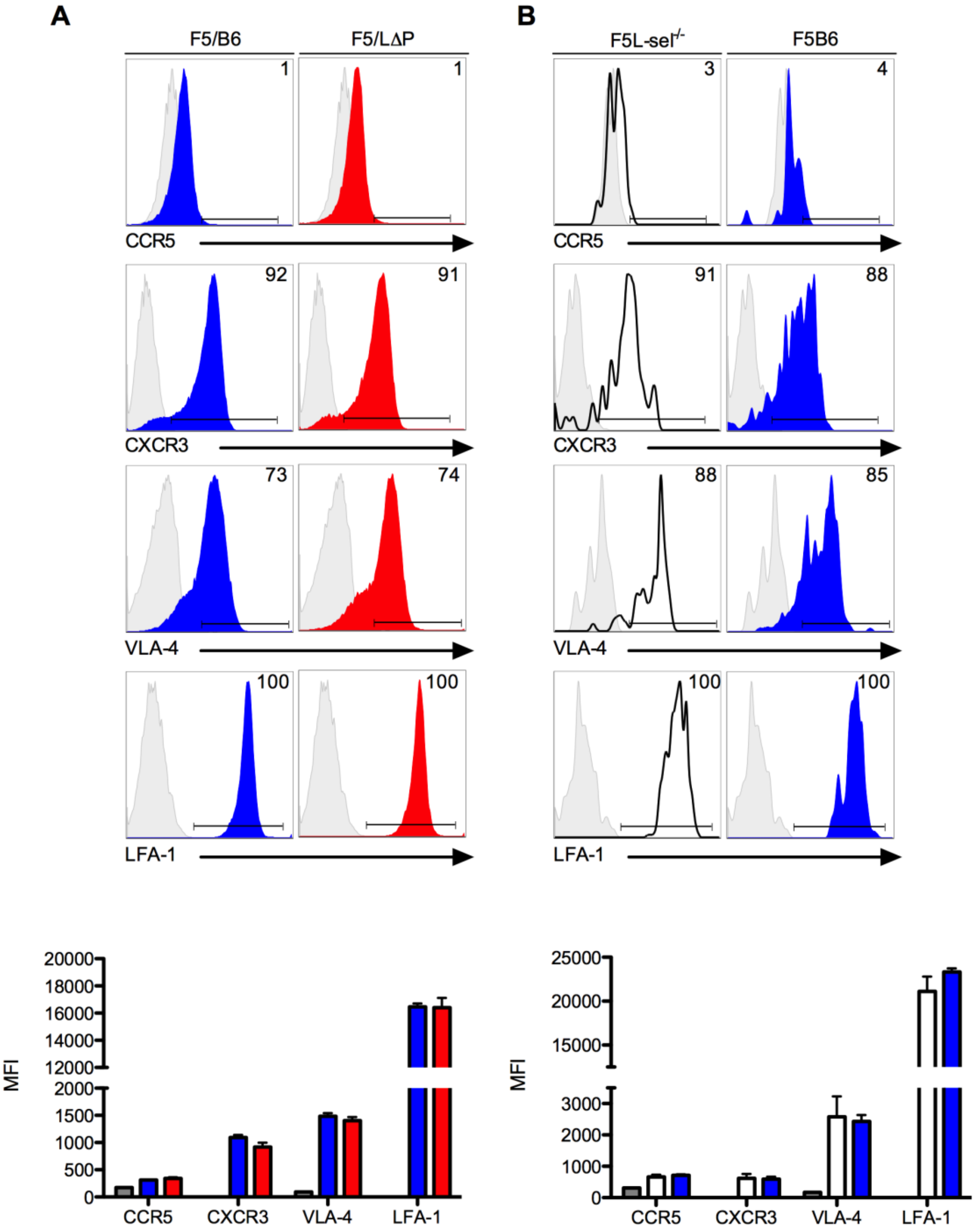
A
F5/B6
F5/LΔP
1
1
CCR5
92
91
CXCR3
73
74
VLA-4
100
100
LFA-1
B
F5L-sel-/-
F5B6
3
4
CCR5
91
88
CXCR3
88
85
VLA-4
100
100
LFA-1
MFI
20000
18000
16000
14000
12000
2000
1500
1000
500
0
CCR5
CXCR3
VLA-4
LFA-1
MFI
25000
20000
15000
3000
2000
1000
0
CCR5
CXCR3
VLA-4
LFA-1

Figure S2, Related Figure 3: L-selectin does not affect expression of inflammation associated homing molecules on virus specific CD8$^+$ T cells. (A) F5/B6 (blue) and F5/LΔP (red) or **(B)** F5L-sel$^{-/-}$ (open) and F5B6 (blue) CD8$^+$ T cells were transferred into naïve B6 mice and 24 hours later mice given vaccNP i.p. Spleens were collected 5 day p.i. and donor cells were identified as in Fig. 2. Representative histograms show percentage positive CCR5, CXCR3, VLA-4 (CD49d) and LFA-1 (CD11a) by donor CD8$^+$ T cells. Bar charts show mean $\pm$ SEM MFI (n=4).

Table S1, Related to Experimental Procedures: Mice

Mice	F5 genotype	Endogenous L-selectin genotype	Transgenic LΔP genotype	CD90 genotype	Fold increase L-selectin expression over wildtype
F5/B6	+/-	+/+	-	CD90.2	1.0
F5/LΔP	+/-	-/-	+/-	CD90.2	1.5[a]
F5/ CD90.1B6	+/-	+/+		CD90.1/CD90.2	1.0
F5B6	+/+	+/+	-	CD90.2	1.0
F5LΔP	+/+	-/-	+/+	CD90.2	3.0[a]
F5L-sel$^{-/-}$	+/+	-/-	-	CD90.2	No expression
LΔP	-/-	-/-	+/+	CD90.2	3.0[a]
L-sel$^{-/-}$	-/-	-/-	-/-	CD90.2	No expression
C57BL/6 (B6)	-/-	+/+	-/-	CD90.2	1.0

[a] Data from (Galkina et al., 2007)

Supplemental Experimental Procedures

Flow Cytometry

Single cell isolate stained with PE-conjugated H2-D^b NP68-tetramer (Influenza H17 derived nucleoprotein 366-374 ASNENMDAM) or H-2D^b NP34-tetramer (Influenza A-PR8–34 nucleoprotein 366-374 ASNENMETM) at 37°C, followed by surface stains at 4°C with FITC-anti Vβ11 (clone: KT-11, Biolegend), PerCPCy5.5-anti CD8 (53-6.7, Biolegend), PECy7-anti CD62L (MEL-14, Biolegend), APC-anti CD69 (H1.2F3, Biolegend), APCCy7-anti CD44 (IM7, Biolegend), Pacific Blue-anti CD90.2/Thy1.2 (53-2.1, Biolegend), AlexaFluor 488-anti CD90.2 (30-H12, Biolegend) and intracellular stains with PerCPCy5.5-anti IFN-γ (XMG1.2, Biolegend), PE-Cy7-anti TNF-α (TN3-19, eBioscience), eFluor-450-anti IL-2 (JES6-5H4, eBiosceince) FITC anti-granzymeB (GB-11, Biolegend), Biotinylated-anti CCR5 (HM-CCR5 7A4, eBioscience), APC-anti CXCR3 (CXCR3-173, eBioscience), PE-anti CD49d (PS/2, Southern Biotechnology), PECy7-anti CD11a (M17/4, Biolegend). Fluorescence minus one (FMO) was used for non-specific, background staining. Cells were fixed and analyzed on a BD FACS Canto II flow cytometer and counted using Cytocount beads (DAKO). Figures were prepared using FlowJo software.

Short term homing assays

For *in vitro* activated CD8+ T cell experiment, F5LΔP and F5L-$sel^{-/-}$ $CD8^+$ T cells were purified and then re-suspended in complete medium (DMEM supplemented with 10% FCS, penicillin-streptomycin, L-glutamine, non-essential amino-acids and 2-β mercaptoethanol) and plated at 2 x 10^6 cells/well in 24-well plates (Nunclon). Splenocytes from F5B6 mice were pulsed with 5 µg/ml NP68 peptide (Peptide Synthetics) for 1 hour at 37^oC, irradiated at 3000 centrigrays, 6 x10^6 splenocytes added per well and plates incubated at 37°C in 5% CO_2. Fresh complete medium supplemented with 360 IU/ml hrIL-2 was added after 2 days. Cells were harvested at day 7, mixed 1:1 and a total of 5.58 x 10^6 cells (2.76 x 10^6 F5LΔP and 2.82 x 10^6 F5L-$sel^{-/-}$ determined using Cytocount beads) injected i.v. into 4 vaccNP infected $CD90.1^+$ B6 mice. After 3 hours, ovaries, ovdLNs, axillary lymph nodes, inguinal lymph nodes, mediastinal lymph node, spleen, peripheral blood were harvested, stained for CD8, CD90.2 and L-selectin and analyzed by flow cytometry for numbers and L-selectin expression on donor $CD8^+$, $CD90.2^+$ cells. For in vivo activated CD8+ T cells, F5LΔP and F5L-$sel^{-/-}$ mice were infected with 2x10^6 vaccNP i.p., At day 5 post infection, mice were euthanized and activated $CD8^+$ T cells were isolated from spleen, mixed 1.16:0.84 and a total of 13.68 x 10^6 cells (5.8 x 10^6 F5LΔP and 7.87 x 10^6 F5L-$sel^{-/-}$) injected into 4 vaccNP infected $CD90.1^+$ B6 mice.

L-selectin inhibition and T cell proliferation in virus-infected mice

To inhibit L-selectin function, 250 µg of monoclonal antibody to mouse L-selectin (MEL-14) or rat IgG2a isotype control antibodies MAC193 (Price et al., 1997) or MAC 219 (Forsyth et al., 2000) were injected i.v.and/or i.p. either once or daily commencing 52 h post infection. The thymidine analogue 5-ethynyl-2'deoxyuridine (EdU; Life Technology,) was re-suspended in saline and 1mg injected i.p. 24 hour prior to tissue harvest.

rIL-2

Human recombinant interleukin-2 (hrIL-2) (Proleukin, Novartis Pharma,) was diluted in saline and $RAG1^{-/-}$ mice injected daily with 150,000 IU hrIL-2.

Supplemental References

Forsyth, I.A., Hutchings, A., and Butcher, G.W. (2000). A panel of monoclonal antibodies to ovine placental lactogen. J Endocrinol *165*, 435-442.

Galkina, E., Florey, O., Zarbock, A., Smith, B.R., Preece, G., Lawrence, M.B., Haskard, D.O., and Ager, A. (2007). T lymphocyte rolling and recruitment into peripheral lymph nodes is regulated by a saturable density of L-selectin (CD62L). Eur J Immunol *37*, 1243-1253.

Price, A.A., Cumberbatch, M., Kimber, I., and Ager, A. (1997). Alpha 6 integrins are required for Langerhans cell migration from the epidermis. J. Exp. Med. *186*, 1725-1735.
