# Supplementary material for: L-selectin Is Essential for Delivery of Activated CD8+ T Cells to Virus-Infected Organs for Protective Immunity
Source: Cell Rep. 2016 Jan 21;14(4):760–71. doi: 10.1016/j.celrep.2015.12.090 (PMC4742564; doi:10.1016/j.celrep.2015.12.090)
Supplement: Document S2. Article plus Supplemental Information [file mmc2.pdf]

Cell Reports

Article

L-selectin Is Essential for Delivery of Activated CD8$^+$ T Cells to Virus-Infected Organs for Protective Immunity

Graphical Abstract

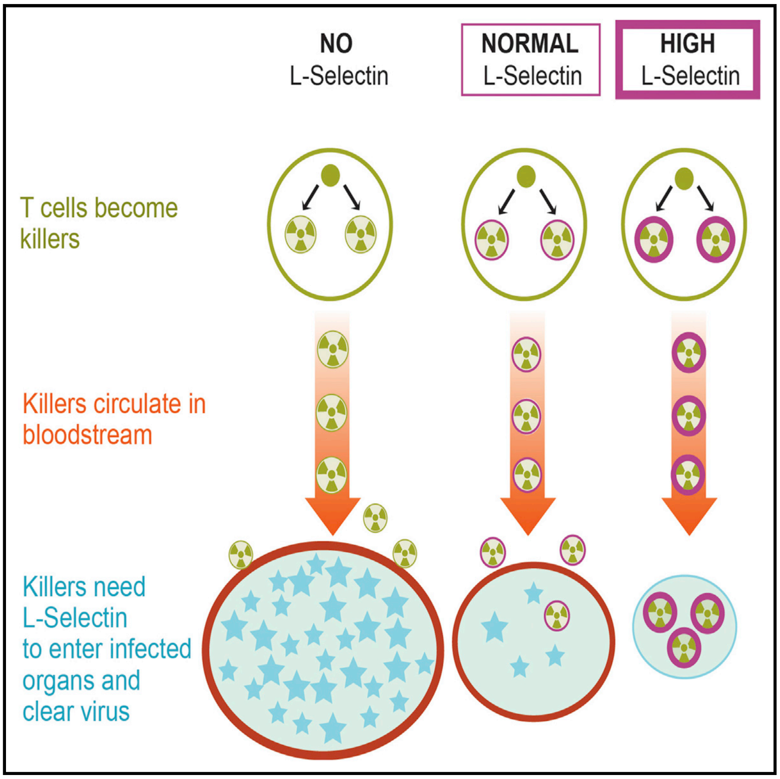

Highlights

- L-selectin is re-expressed on activated CD8$^+$ T cells exiting lymph nodes
- L-selectin does not regulate priming, differentiation, or function of cytotoxic T lymphocytes
- Entry of activated CD8$^+$ T cells into virus-infected tissues is L-selectin dependent
- The level of cell-surface L-selectin determines the extent of anti-viral immunity

Authors

Rebar N. Mohammed, H. Angharad Watson, Miriam Vigar, Julia Ohme, Amanda Thomson, Ian R. Humphreys, Ann Ager

Correspondence

agera@cardiff.ac.uk

In Brief

Lymphocyte migration is a key step in the host's immune response against viruses. Mohammed et al. demonstrate that re-expressed L-selectin/CD62L on primed virus-specific CD8$^+$ T cells plays a pivotal role in their recruitment from the bloodstream into virus-infected tissues for protective immunity.

Mohammed et al., 2016, Cell Reports *14*, 760–771
February 2, 2016 ©2016 The Authors
http://dx.doi.org/10.1016/j.celrep.2015.12.090

L-selectin Is Essential for Delivery of Activated CD8$^+$ T Cells to Virus-Infected Organs for Protective Immunity

Rebar N. Mohammed,[1,2] H. Angharad Watson,[1,2] Miriam Vigar,[1,2] Julia Ohme,[1,2] Amanda Thomson,[1,2] Ian R. Humphreys,[1,2] and Ann Ager[1,2,*]
[1]Institute of Infection and Immunity
[2]Systems Immunity Research Institute
School of Medicine, Cardiff University, Cardiff CF14 4XN, UK
*Correspondence: agera@cardiff.ac.uk
http://dx.doi.org/10.1016/j.celrep.2015.12.090
This is an open access article under the CC BY license (http://creativecommons.org/licenses/by/4.0/).

SUMMARY

Cytotoxic CD8$^+$ T lymphocytes play a critical role in the host response to infection by viruses. The ability to secrete cytotoxic chemicals and cytokines is considered pivotal for eliminating virus. Of equal importance is how effector CD8$^+$ T cells home to virus-infected tissues. L-selectin has not been considered important for effector T cell homing, because levels are low on activated T cells. We report here that, although L-selectin expression is down-regulated following T cell priming in lymph nodes, L-selectin is re-expressed on activated CD8$^+$ T cells entering the bloodstream, and recruitment of activated CD8$^+$ T cells from the bloodstream into virus-infected tissues is L-selectin dependent. Furthermore, L-selectin on effector CD8$^+$ T cells confers protective immunity to two evolutionally distinct viruses, vaccinia and influenza, which infect mucosal and visceral organs, respectively. These results connect homing and a function of virus-specific CD8$^+$ T cells to a single molecule, L-selectin.

INTRODUCTION

CD8$^+$ T cells play a prominent role in the host response to infection with a variety of pathogens, most notably, viruses. The activation, proliferation, and differentiation of naive CD8$^+$ T cells into effector cytotoxic T cells have been studied extensively in mice in response to a number of different viruses, using various routes of inoculation (Fung-Leung et al., 1991; Goulding et al., 2014; Zinkernagel and Doherty, 1979). The consensus from these studies is that effector CD8$^+$ T cells are generated from naive CD8$^+$ T cells inside lymphoid organs draining the site of virus inoculation. Following exit from the lymph node (LN) and entry into the bloodstream, effector CD8$^+$ T cells migrate to virus-infected tissues in response to inflammatory stimuli produced by the virus to clear/resolve the infection. Effector CD8$^+$ T cells also migrate to many different non-lymphoid organs that are not infected by virus (Masopust et al., 2004). The widespread dissemination of virus-specific CD8$^+$ T cells to tissues in which they are not needed may limit the number available to clear virus from infected organs and thereby reduce their efficacy. Extensive studies have elucidated the different mechanisms that effector CD8$^+$ T cells use to eliminate virus (Zhang and Bevan, 2011). However, the recruitment of virus-specific effector CD8$^+$ T cells from the bloodstream into tissues in the resolution of a primary infection is equally important to understand.

Intravital imaging has revealed that a key event in the selection of blood-borne leukocytes for recruitment into tissues is their capture, rolling, and arrest on the inside walls of blood vessels. This depends on co-ordinated signaling of different types of adhesion molecule, such as selectins and integrins, as well as chemokine receptors following engagement by their respective ligands on blood vessel endothelial cells. Virus-specific effector CD8$^+$ T cells upregulate the expression of a number of adhesion molecules known to regulate the recruitment of activated or effector T lymphocytes into sites of inflammation, including P-selectin glycoprotein ligand (PSGL)-1, CD44, and the integrins LFA-1 and VLA-4 (Austrup et al., 1997; DeGrendele et al., 1997; Mora and von Andrian, 2006; Oehen and Brduscha-Riem, 1998; Siegelman et al., 2000; Lefrançois and Marzo, 2006; Liu et al., 2006). Depending on whether virus inoculation is via the skin or the mucosa, upregulation of skin homing molecules such as cutaneous lymphocyte antigen (CLA) or the mucosal homing receptor $\alpha 4\beta 7$ integrin could also impart tissue-specific homing properties to virus-specific effector CD8$^+$ T (Liu et al., 2006). However, direct evidence for any of the homing-associated molecules expressed by activated virus-specific CD8$^+$ T cells regulating their recruitment from the bloodstream into infected tissues is lacking. In fact, a recent study found that the chemokine receptor CXCR3, which is widely implicated in the homing of interferon (IFN)-γ secreting CD8$^+$ T cells, had no role in the recruitment of virus-specific CD8+ T cells from the bloodstream into infected skin (Hickman et al., 2015).

A striking feature of virus-specific effector CD8$^+$ T cells, regardless of the route of virus inoculation, is downregulation of the adhesion molecule leukocyte-selectin (L-selectin)/CD62L. Low expression of L-selectin on effector T cells (Kaech et al., 2002;

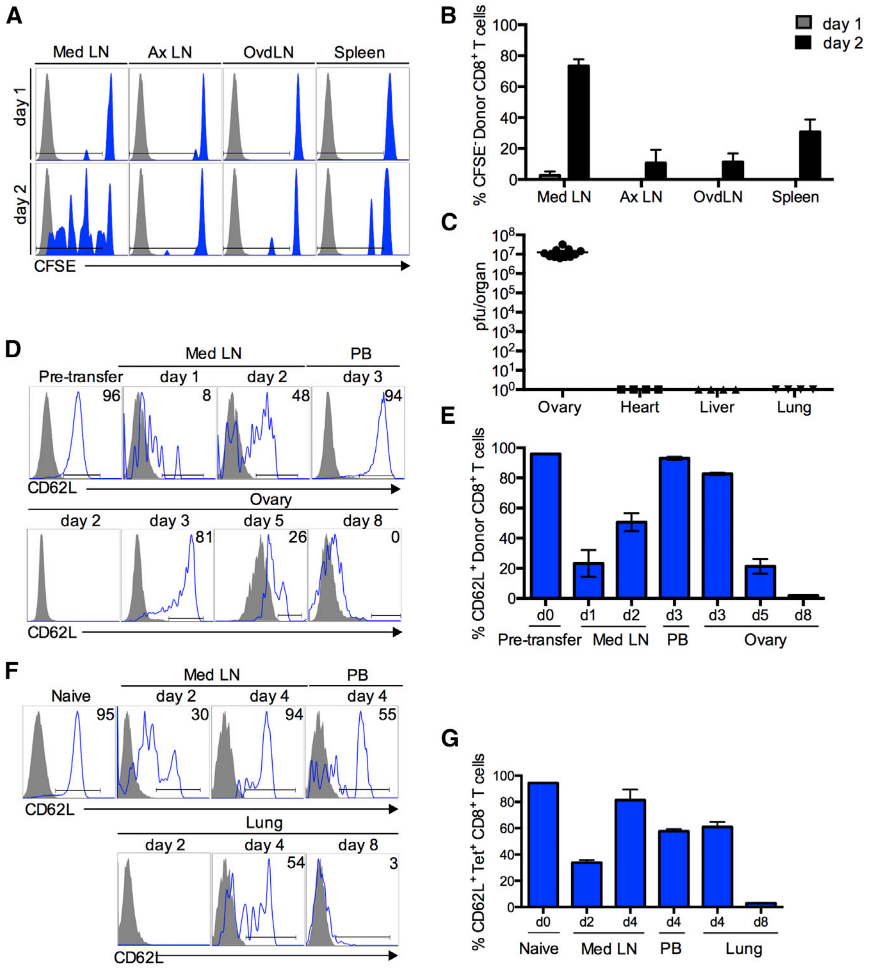

Figure 1. Cyclical Expression of L-selectin during the Development of Virus-Specific Effector CD8⁺ Cells In Vivo

(A) CFSE-labeled F5/B6 CD90.2 CD8⁺ T cells were transferred i.v. to naive CD90.1 B6 mice, and 24 hr later, mice were given vaccNP i.p. Lymphoid organs were harvested 1 and 2 days after virus challenge, and donor cells were analyzed for dilution of CFSE in mediastinal LN (Med LN), axillary LN (Ax LN), and ovary draining LN (ovDLN), and spleen (blue) compared with control unlabeled cells (gray).

(B) Bar chart shows means ± SEM for percentage of CFSE-diluted donor cells (n = 3).

(C) Naive B6 mice were given 2 × 10^6 pfu of vaccNP i.p., and ovary, heart, lung, and liver tissues were collected after 5 days. Virus titers in homogenized tissues were determined by plaque assay. Symbols indicate individual mice.

(D) Naive F5/B6 CD90.2 CD8⁺ T cells were transferred i.v. to naive CD90.1 B6 mice, and 24 hr later, mice were injected with vaccNP. Representative histograms show L-selectin expression on donor CD8⁺ T cells in mediastinal LN (Med LN), peripheral blood (PB), and ovary.

(E) Bar chart shows means ± SEM for percentage of L-selectin-positive donor cells (n = 4). d, day.

(F) Naive B6 mice were infected intranasally with 50 ffu influenza A virus (H1N1, PR8). Representative histograms show L-selectin expression on PR8-specific tetramer (tet)-positive CD8⁺ T cells in mediastinal LN, PB, and lungs.

(G) Bar chart shows means ± SEM for percentage of L-selectin-positive PR8-specific CD8⁺ T cells (n = 4).

Mobley and Dailey, 1992; Richards et al., 2008), together with homing studies, have suggested that L-selectin is not an important homing molecule for sites of inflammation (Austrup et al., 1997; Hirata et al., 2002; Mobley and Dailey, 1992; Mora and von Andrian, 2006). Importantly, however, the recruitment of T cells to skin allografts and peritoneal inflammation are significantly reduced in L-selectin-deficient mice (Tang et al., 1997; Tedder et al., 1995). L-selectin expression rapidly cycles between high and low levels on in-vitro-activated T cells (Chao et al., 1997). Variable expression of L-selectin during T cell differentiation may, therefore, contribute to the conflicting data on whether L-selectin is an important homing molecule on effector T cells in vivo.

In this study, we have used two evolutionary distinct viruses, vaccinia and influenza, that infect mucosal and visceral organs, respectively, and determined the role of L-selectin in regulating the recruitment and function of effector CD8⁺ T cells. We demonstrate that, following activation in LNs draining the site of inoculation, L-selectin promotes the recruitment of effector CD8⁺ T cells to two different sites of virus replication: the lungs and ovaries. Importantly, L-selectin is required for effective control of virus replication in these organs. These results connect homing and a function of virus-specific CD8⁺ T cells to a single molecule, L-selectin.

RESULTS

Cyclical Expression of L-selectin during Activation of Virus-Specific Effector CD8⁺ T Cells In Vivo

Recombinant vaccinia virus expressing nucleoprotein peptide $NP_{366–374}$ (vaccNP) (Townsend et al., 1986) and CD8⁺ T cells expressing cognate F5 T cell receptor were used to determine the levels of L-selectin expression at different stages of T cell activation in vivo. First, the sites of T cell activation following intraperitoneal administration of vaccNP were determined. CFSE-labeled, naive F5/B6 CD8⁺ T cells were injected into C57BL/6 (B6) mice, vaccNP was administered 24 hr later, and lymphoid organs were analyzed for CFSE-labeled donor T cells at 1 and 2 days postinfection (p.i.) (Figures 1A and 1B). Dilution of CFSE was not detected until the second day following virus administration and was largely restricted to the mediastinal LN. T cell priming was low in LNs that do not drain the peritoneal cavity (<10%) and slightly higher in the spleen. As described previously, high virus titers were found in the ovaries, but in contrast to the parental Western Reserve strain (Zhao et al., 2011), virus was not detected in other visceral organs (Figure 1C). Interestingly, there was no evidence of T cell priming in LNs draining the ovary (ovdLNs) at days 1 and 2 p.i. Therefore, CD8⁺ T cell

OPEN
ACCESS
CellPress

activation following intraperitoneal administration of vaccNP occurs within the first 2 days and is largely restricted to the mediastinal LN that drains the site of virus inoculation.

Next, we investigated changes in cell-surface levels of L-selectin/CD62L following T cell activation in mediastinal LN and recruitment to virus-infected ovaries (Figures 1D and 1E). Naive $CD8^+$ T cells express high levels of L-selectin. Following virus administration, L-selectin is rapidly downregulated within 24 hr. L-selectin re-expression begins during the second day of infection, when activated $CD8^+$ T cells are still resident in mediastinal LNs. By day 3, activated $CD8^+$ T cells are detectable in the bloodstream, and the majority (93% ± 1%) have re-expressed L-selectin. $CD8^+$ T cells start to infiltrate virus-infected ovaries on day 3 p.i., at which time, the majority of cells still express L-selectin (83% ± 1% positive). L-selectin expression gradually declines to 21% ± 5% and 2% ± 0.1% positive at days 5 and 8 p.i., respectively. Cyclical expression of L-selectin was also seen on endogenous, polyclonal virus-specific $CD8^+$ T cells following intranasal (i.n.) infection of B6 mice with influenza A PR8 strain (Figures 1F and 1G). L-selectin was downregulated and re-expressed at days 2 and 4 p.i., respectively, in the draining mediastinal LN, and L-selectin expression on virus-specific $CD8^+$ T cells in the bloodstream recruited to the lungs gradually declined from 60% positive at day 4 to less than 5% positive at day 8 p.i. These cyclical changes in L-selectin mirror the time course of early ectodomain shedding, subsequent re-expression, and transcriptional silencing of L-selectin reported in vitro (Chao et al., 1997) and raise the possibility that re-expressed L-selectin could control the homing of activated $CD8^+$ T cells to virus-infected tissues.

Recruitment of $CD8^+$ T Cells to Virus-Infected Organs Is L-selectin Dependent

We hypothesized that, if T cell recruitment to virus-infected tissues is L-selectin dependent, T cells expressing higher than normal levels of L-selectin will be enriched and L-selectin-deficient T cells will be decreased. To test this hypothesis, naive F5 $CD8^+$ T cells expressing either physiological levels of wild-type L-selectin (F5/B6) or increased levels of L-selectin (F5/LΔP) (Figures 2A and 2B; Table S1) were transferred into B6 mice, the mice were challenged the following day with vaccNP and ovaries, and ovdLNs and spleens were analyzed for donor cells (Figure 2C). F5/B6 cells were found in ovaries at days 5, 8, and 12 p.i. (Figure 2D). The numbers did not change during the course of the infection and, as described in Figure 1C, L-selectin expression gradually fell. The number of F5/LΔP cells in the ovaries were 5- and 10-fold higher than F5/B6 cells at days 5 and 8, respectively (Figure 2D). By day 12, F5/LΔP T cells had fallen in number and were not significantly different from F5/B6 cells (Figure 2D), suggesting that expression of L-selectin, which is maintained on all activated F5/LΔP cells (Figure 2C) (Richards et al., 2008), does not improve survival or increase retention of virus-specific $CD8^+$ T cells at the site of infection. At day 5 p.i., F5/LΔP $CD8^+$ T cells were enriched in ovdLNs, although this was not significant and less than that seen in ovaries (Figure 2D). The numbers of F5 $CD8^+$ T cells expressing wild-type or increased levels of L-selectin (LΔP) in the spleen peaked at day 8 and were 50- to 1,000-fold higher than in ovaries or ovdLNs at all times analyzed. However, there was no enrichment of F5/LΔP over F5/B6 at any time point during virus infection, since lymphocyte entry to the spleen is not L-selectin dependent (Figure 2D) (Arbonés et al., 1994).

To determine the role of endogenous L-selectin, naive $CD8^+$ T cells either sufficient (F5B6) or deficient (F5L-sel$^{-/-}$) in L-selectin were used. L-selectin expression was not required for entry into mediastinal LN (Figure S1), and L-selectin expression had no effect on T cell activation, since L-selectin-deficient and L-selectin-sufficient $CD8^+$ T cells were equally primed as measured by onset of proliferation following virus administration (Figure S1). However, the number of F5L-sel$^{-/-}$ $CD8^+$ T cells recruited to vaccinia-infected ovaries was markedly lower than that of wild-type cells at day 5 p.i. (Figure 2E). There were also reduced numbers of F5L-sel$^{-/-}$ $CD8^+$ T cells in ovdLNs. Similar numbers of F5 $CD8^+$ T cells, sufficient or deficient in L-selectin, were found in the spleen (Figure 2E). L-selectin expression also controlled the recruitment of endogenously generated influenza-specific $CD8^+$ T cells to infected lungs. As shown in Figure 2F, the number of PR8-specific $CD8^+$ T cells in the lungs of polyclonal LΔP mice was 2-fold higher than that of B6 mice, whereas similar numbers were found in the spleens.

These results, using two evolutionarily distinct viruses targeting different internal organs, demonstrate that the level of L-selectin on activated $CD8^+$ T cells controls recruitment to sites of virus infection.

L-selectin Does Not Influence Priming, Activation, and Differentiation of Virus-Specific $CD8^+$ T Cells

Differences in recruitment of virus-specific $CD8^+$ T cells sufficient and deficient in L-selectin raised the possibility that L-selectin affects the priming of $CD8^+$ T cells, which, in turn, controls the number of effector cells generated. Therefore, we determined the kinetics of T cell activation and differentiation during the course of virus infection. The early activation antigen CD69 was upregulated by day 1 on wild-type F5/B6 $CD8^+$ T cells in mediastinal LN following vaccNP administration, and the kinetics of CD69 upregulation on F5/LΔP $CD8^+$ T cells were similar (Figure 3A), suggesting that increased levels of L-selectin did not alter interactions with antigen-presenting cells at the site of priming. CD69 was equally upregulated on L-selectin-deficient and L-selectin-sufficient $CD8^+$ T cells in mediastinal LNs following i.n. administration of influenza virus, confirming that L-selectin expression is not required to prime naive $CD8^+$ T cells to virus-derived peptides (Figure 3B).

To determine whether L-selectin affects the differentiation of virus-specific $CD8^+$ T cells during the course of infection, expression of the late activation antigen CD44 on virus-specific $CD8^+$ T cells in virus-infected organs, LNs, and spleen was measured. CD44 was upregulated to a similar extent and with identical kinetics on T cells expressing wild-type L-selectin (B6), expressing increased levels of L-selectin (LΔP), or deficient in L-selectin (L-sel$^{-/-}$); on donor F5 T cells following intraperitoneal vaccNP administration (Figures 3C and 3D) and on endogenously generated influenza-specific $CD8^+$ T cells following i.n. influenza administration (Figure 3E).

All donor T cells were fully activated (>90% CD44 positive) by day 5 p.i., apart from in the spleen, where full activation was not

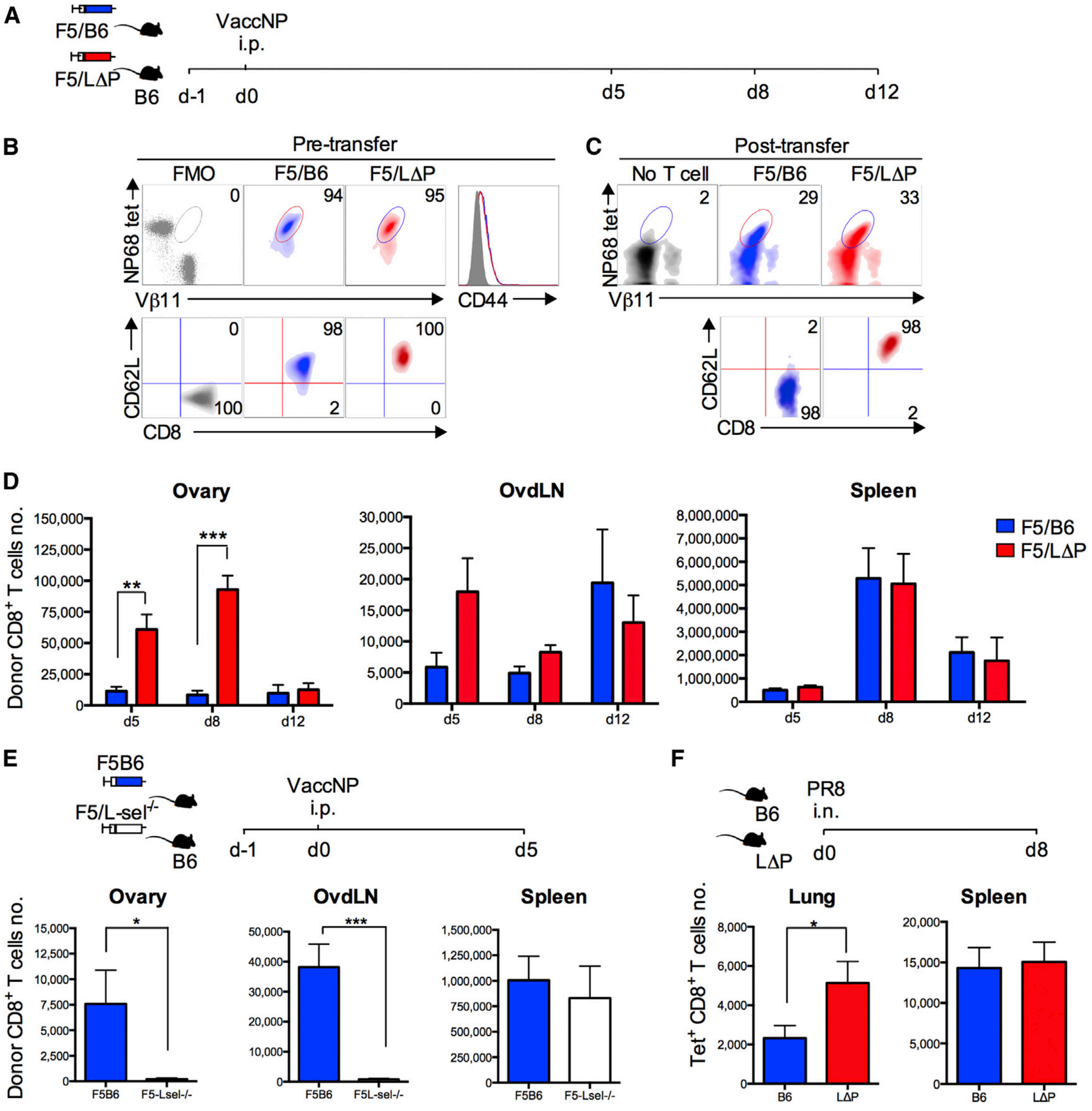

Figure 2. L-selectin-Dependent Recruitment of CD8+ T Cells to Virus-Infected Tissues

(A and B) Experimental plan (A) and density plots (B) showing F5 TCR expression on pre-transferred naive F5/B6 (blue) and F5/LΔP (red) $CD8^+$ T cells using NP68 tetramer (tet) and Vβ11 staining, and fluorescence minus NP68 tetramer and Vβ11 was used as fluorescence minus one (FMO). Overlaid histograms show CD44 expression, and gray histogram shows CD44 FMO. Density plots show L-selectin (CD62L) expression on $CD8^+$ T cells, and gray density plot shows CD62L FMO. In (B), percentage of positive cells is shown in the top right corners. d, day.

(C) Density plots show CD8, NP68 tetramer, and Vβ11 triple-positive F5/B6 and F5/LΔP donor cells post-transfer and background staining in mice that did not receive F5 $CD8^+$ T cells (No T cell). Representative density plots and percentages of donor F5/B6 and F5/LΔP $CD8^+$ T cells and L-selectin expression on donor T cells in ovaries are shown.

(D) Total numbers of donor F5/B6 or F5/LΔP $CD8^+$ T cells at days 5, 8, and 12 post-vaccNP infection in ovary, ovdLN, and spleen. Two-way ANOVA with Bonferroni post-test analysis.

(E) Total numbers of donor F5 $CD8^+$ T cells expressing wild-type L-selectin (F5B6) or deficient in L-selectin (F5L-sel$^{-/-}$) at day 5 p.i. in ovary, ovdLN, and spleen.

(F) Total numbers of endogenous, influenza-specific $CD8^+$ T cells at day 8 p.i. with influenza A virus (PR8, H1N1) in lungs and spleens of B6 and LΔP mice. i.n., intranasal.

Bar charts show means ± SEM. $^*p < 0.05$; $^{**}p < 0.005$; $^{***}p < 0.0001$.

seen until day 8 p.i. (Figure 3C). CD44 has been shown to regulate effector T cell migration to sites of inflammation, but expression levels were similar on F5LΔP, F5B6, and F5L-sel$^{-/-}$ $CD8^+$ T cells throughout the course of virus infection. The expression of other adhesion molecules that regulate effector T cell migration, such as LFA-1 and VLA-4, as well as inflammation-associated chemokine receptors, CCR5 and CXCR3, were also equally upregulated on activated F5LΔP, F5B6, and F5L-sel$^{-/-}$ $CD8^+$ T cells (Figure S2). Together, these results demonstrate clearly that L-selectin does not influence $CD8^+$ T cell priming in

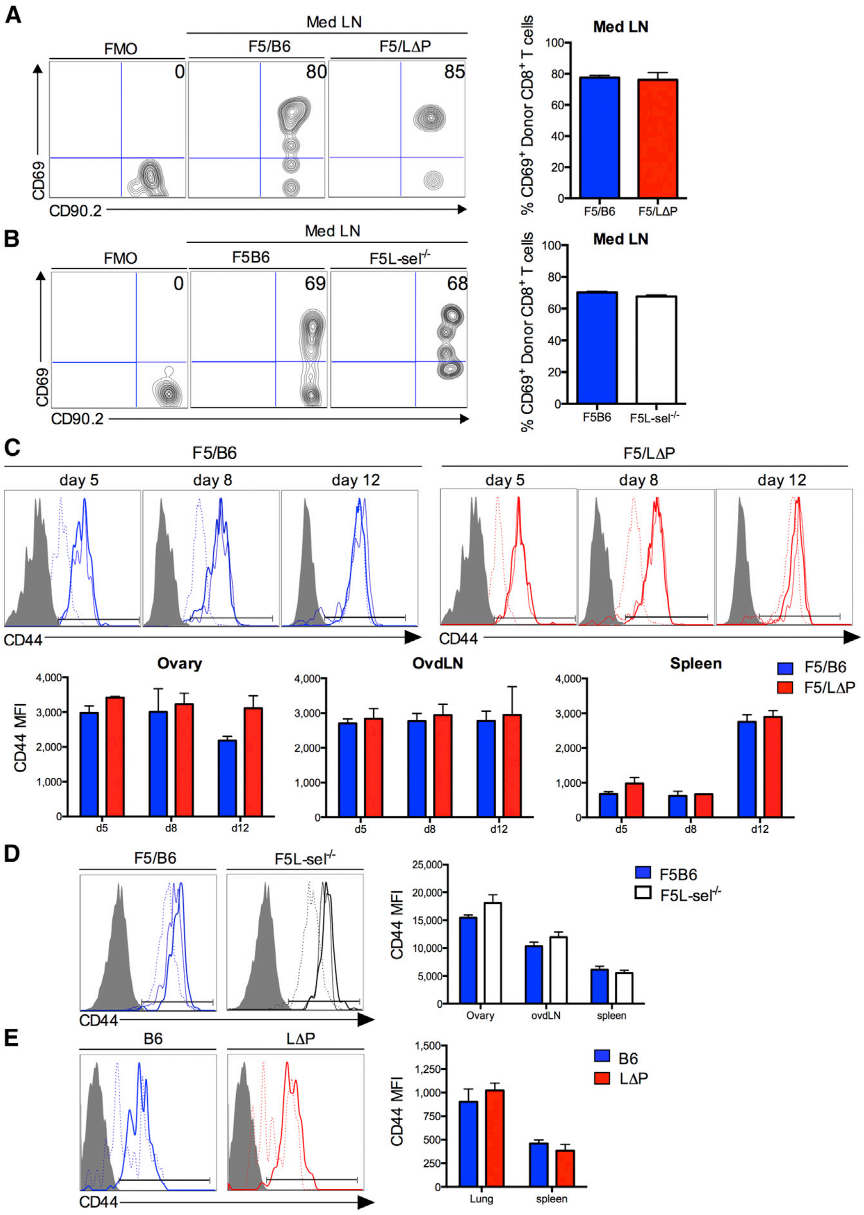

Figure 3. L-selectin Expression Does Not Affect the Activation or Differentiation of CD8$^+$ T Cells following intraperitoneal or Intranasal Virus Administration

(A and B) Representative plots show (A) percentages of CD69-positive F5/B6 and F5/LΔP CD8$^+$ T cells in mediastinal LN (Med LN) (day 1 p.i. with vaccNP) and (B) percentages of CD69-positive F5B6 and F5L-sel$^{-/-}$ CD8$^+$ T cells in mediastinal LN at day 2 p.i. with H17 influenza virus. Bar charts show means ± SEM.

(C and D) Representative plots show (C) CD44-positive F5/B6 and F5/LΔP at days 5, 8, and 12 p.i. with vaccNP and (D) F5B6 and F5L-sel$^{-/-}$ CD8$^+$ T cells at day (d) 5 p.i. with vaccNP in ovary (thick line), ovdLNs (thin line), and spleen (dashed line). Bar charts show means ± SEM. CD44 median fluorescence intensity (MFI).

(E) Representative plots show CD44-positive polyclonal influenza-specific CD8$^+$ T cells in lungs (thick line) and spleen (dashed line) of B6 and LΔP mice (day 8 p.i. with PR8 influenza virus). Bar chart shows means ± SEM. CD44 MFI.

mediastinal LNs, the subsequent activation of primed T cells, or the expression of other homing-associated molecules; instead, the results indicate a role for L-selectin in the recruitment of activated CD8$^+$ T cells from the bloodstream into virus-infected tissues.

L-selectin-Dependent Homing of Activated CD8$^+$ T Cells to Virus-Infected Tissues

Two different approaches were used to determine the role of L-selectin in activated CD8$^+$ T cell homing. Based on the site of priming and kinetics of CD8$^+$ T cell activation, naive F5/LΔP CD8$^+$ T cells were allowed to home and be primed before starting treatment with MEL-14, a blocking antibody to L-selectin (Gallatin et al., 1983). Mice were injected with either 250 μg MEL-14 or immunoglobulin G (IgG)2a isotype control 52 hr p.i., and donor cells were analyzed at day 5 (Figures 4A and 4B). Strikingly, MEL-14 reduced the number of donor CD8$^+$ T cells in infected ovaries by 85% (Figure 4C). MEL-14 also reduced donor CD8$^+$ T cells in ovary-draining LNs but had no effect in the spleen (Figure 4C). Furthermore, to test whether L-selectin affects the homing of endogenously produced effector CD8$^+$ T cells, B6 mice were intranasally infected with PR8 influenza virus, and MEL-14 was administered after T cell priming (Figures 4E and 4F). Systemic L-selectin blockade reduced the number of PR8-specific CD8$^+$ T cells in the lungs by 56% at day 4 and by 78% at day 8 p.i., demonstrating a major role for L-selectin in the recruitment of endogenously produced effector CD8+ T cells to influenza-infected tissues (Figure 4G). The activation of virus-specific CD8$^+$ T cells was not influenced by L-selectin inhibition, as indicated by comparable numbers of virus-specific T cells in the mediastinal LN and spleen (Figures 4C and 4G) and comparable CD44 expression in MEL-14 and IgG-treated virus-infected mice (Figures 4D and 4H).

To confirm that the effect of MEL-14 was due to the blockade of L-selectin on virus-specific CD8$^+$ T cells and not due to the blockade of L-selectin on host leukocytes, a competitive short-term homing experiment was conducted using activated CD8$^+$ T cells sufficient and deficient in L-selectin. We used a congenic CD90.1/90.2 system, exploiting constant expression of L-selectin on LΔP effector CD8$^+$ T cells (Figure 2C) (Richards et al., 2008) and used L-selectin expression to distinguish these

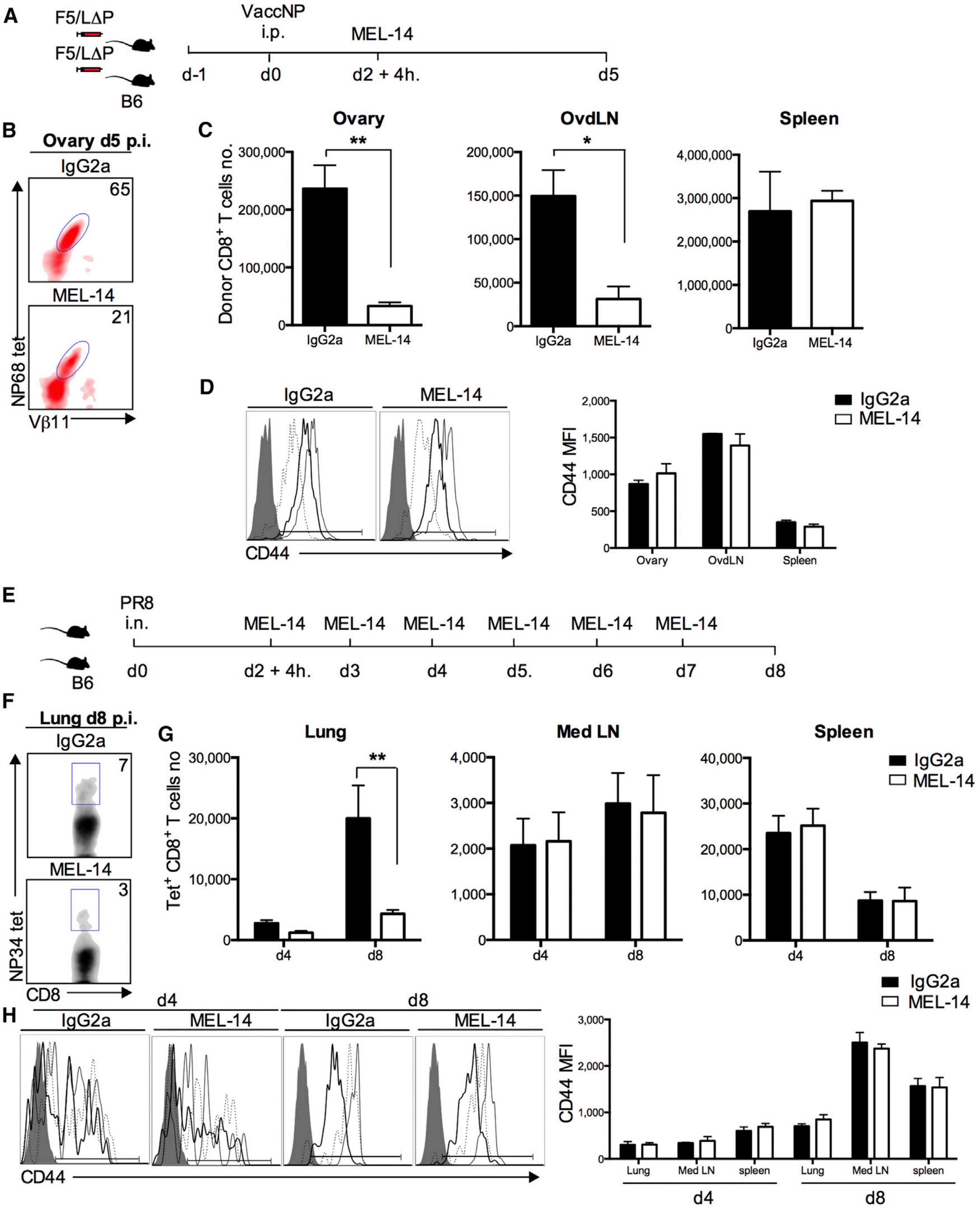

Figure 4. Recruitment of L-selectin-Expressing CD8^{+} T Cells to Virus-Infected Tissues Is Inhibited by Anti-L-selectin Antibodies

(A–C) Presented are: (A) an experimental plan, (B) representative plots showing donor CD8^{+} T cells in ovaries at day (d) 5 p.i. with vaccNP, and (C) numbers of donor cells in ovary, ovdLNs, and spleen of mice given either IgG2a or MEL-14 antibodies.

(D) Representative plots showing CD44-positive donor CD8^{+} T cells in ovary (thick line), ovdLNs (thin line), and spleen (dashed line) at day 5 p.i. with vaccNP in mice given either IgG2a or MEL-14. Bar chart shows means ± SEM. CD44 MFI.

(E–G) Presented are: (E) an experimental plan, (F) representative plots showing influenza-specific CD8^{+} T cells in lungs of mice treated with IgG2a or MEL-14 antibodies at day 8 p.i. with PR8, and (G) numbers of influenza-specific CD8^{+} cells in lung, mediastinal LN (Med LN), and spleen. Two-way ANOVA with Bonferroni post-test.

(H) Representative plots showing CD44-positive influenza-specific CD8+ T cells in lung (thick line), mediastinal LN (thin line), and spleen (dashed line) at days 4 and 8 p.i. in mice injected with either IgG2a or MEL-14. CD44 MFI (days 4 and 8 p.i. with PR8).

Bar charts show means ± SEM. $^{*}p < 0.05$; $^{**}p < 0.005$.

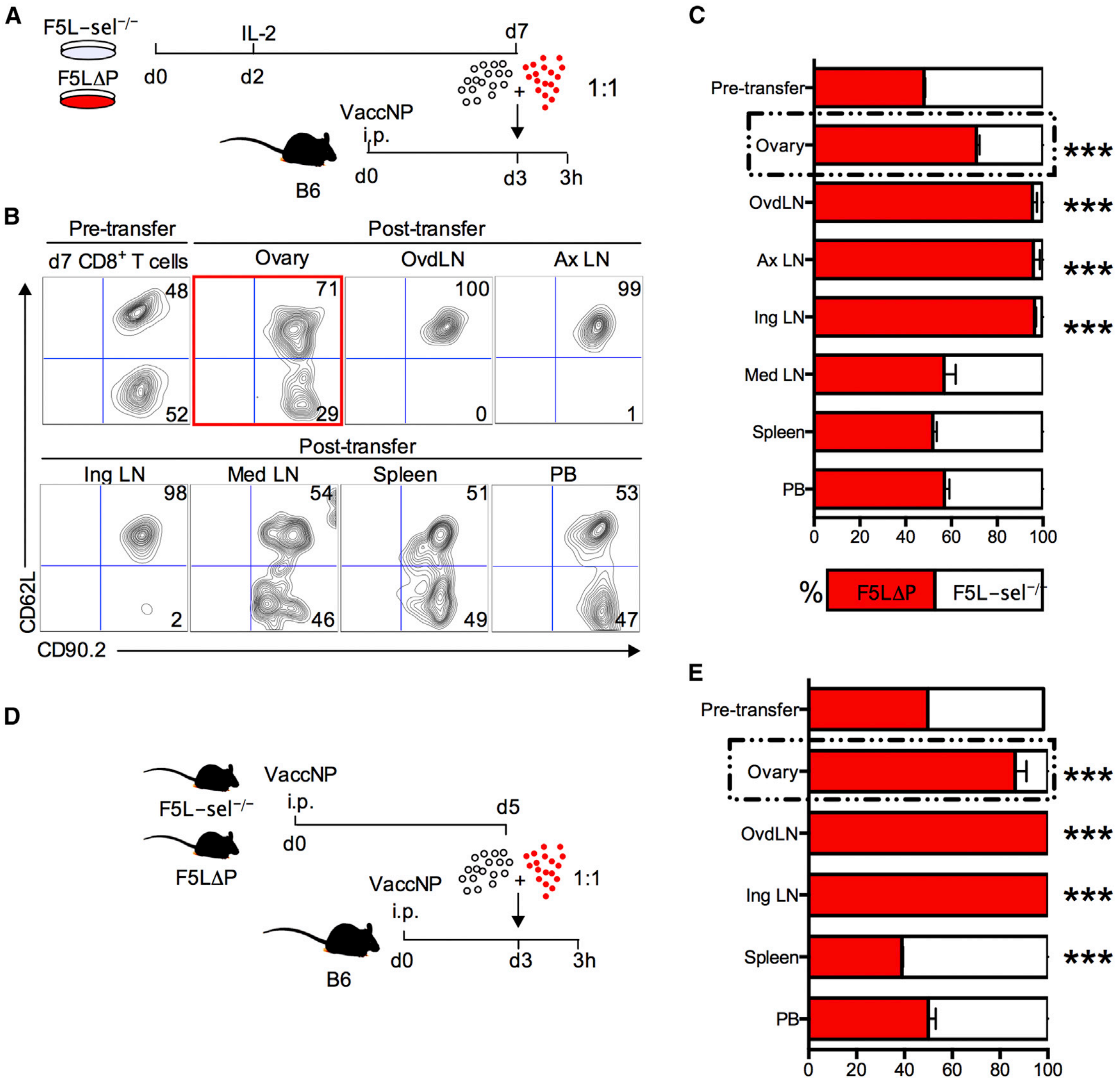

Figure 5. L-selectin Expression by Effector CD8+ T Cells Promotes Homing to Virus-Infected Tissues

(A and B) Presented are: (A) an experimental plan and (B) representative plots showing percentages of a 1:1 mix of in-vitro-activated F5LΔP (CD90.2+CD62L+) and F5L-sel$^{-/-}$ (CD90.2+CD62L−) CD8+ T cells pre- and post-transfer in ovary, ovdLN, axillary LN (Ax LN), inguinal LN (Ing LN), mediastinal LN (Med LN), spleen, and PB.

(C) Bar chart shows means ± SEM.

(D and E) Presented are: (D) an experimental plan and (E) bar chart showing means ± SEM for percentages of in-vivo-activated F5LΔP and F5L-sel$^{-/-}$ CD8+ T cells pre- and post-transfer in ovary, ovdLN, inguinal LN, spleen, and PB. Two-way ANOVA with Bonferroni post-tests.

***$p < 0.0001$.

from L-sel$^{-/-}$ donor T cells in the same host. CD90.1 B6 mice were inoculated with vaccNP and intravenously injected 3 days later with a 1:1 mixture of either in vitro (Figure 5A) or in vivo (Figure 5D) activated F5LΔP and F5L-sel$^{-/-}$ CD8+ T cells. Three hours later, donor cells in different tissue compartments were identified as CD8+, CD90.2+, and either CD62L+ or CD62L− (Figure 5B). L-selectin/CD62L-expressing CD8+ T cells were highly enriched over L-selectin-deficient cells in virus-infected ovaries (Figures 5C and 5E). The ratio of L-selectin-positive and -negative T cells circulating in peripheral blood (PB) remained 1:1, showing that the altered ratio in the ovaries was not simply due to the depletion of L-selectin-deficient T cells from the circulation (Figures 5C and 5E). Interestingly, the enrichment of L-selectin-positive over L-selectin-negative CD8+ T cells was substantially higher, using effector cells generated in vivo rather than effector T cells generated in vitro at 6.2-fold and 2.3-fold, respectively. The majority of donor cells in ovdLNs were L-selectin positive, as in two peripheral LNs known to be L-selectin dependent, the axillary and inguinal LNs (Arbonés et al., 1994). L-selectin-positive T cells were not enriched in the mediastinal LN, which shows clearly that, as found in the case of naive CD8+ T cells (Figure S1), L-selectin is not a dominant homing receptor for

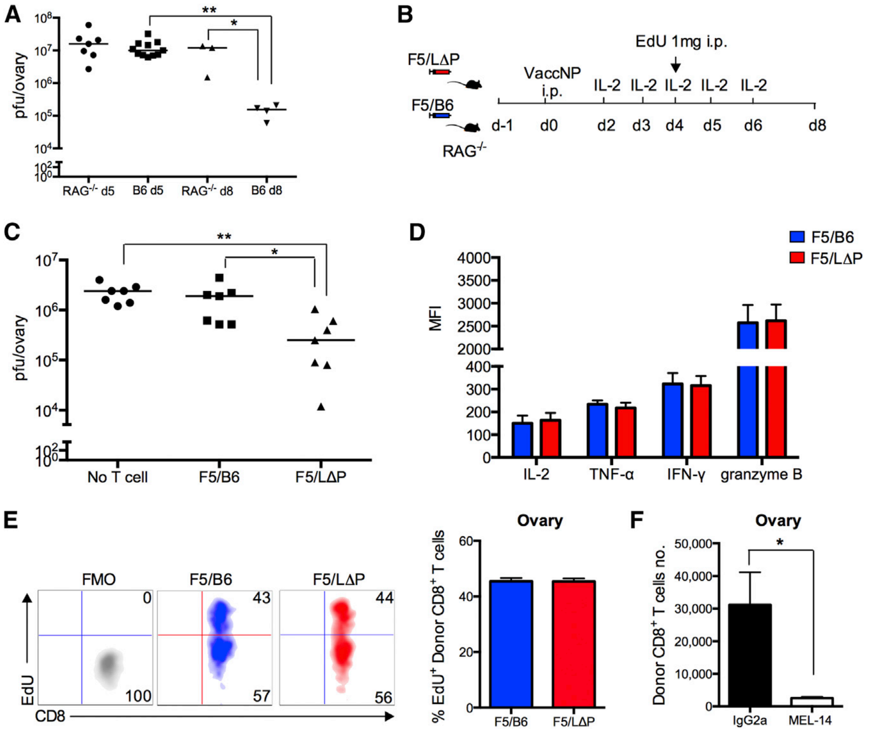

Figure 6. L-selectin Is Essential to Recruit Protective, Virus-Specific $CD8^+$ T Cells during Primary Vaccinia Virus Infection

(A) Virus titers in ovaries of B6 and $RAG1^{-/-}$ mice at day (d) 5 and day 8 p.i. with vaccNP.

(B and C) Presented are: (B) an experimental plan and (C) virus titers in ovaries of $RAG1^{-/-}$ mice supplemented with naive F5/B6 cells, F5/LΔP $CD8^+$ T cells, or no T cells at day 8 p.i. with vaccNP. One-way ANOVA with post-Tukey's multiple comparison test.

(D) Bar chart shows MFI of IL-2, TNF-α, IFN-γ, and granzyme B of donor F5/B6 and F5/LΔP $CD8^+$ T cells in ovaries at day 5 p.i. with vaccNP.

(E) Representative density plots show incorporation of EdU dye into the DNA of proliferating donor F5/B6 and F5/LΔP $CD8^+$ T cells in the ovary at day 5 p.i. with vaccNP.

(F) Bar chart shows number of donor F5/LΔP $CD8^+$ T cells in ovaries of $RAG1^{-/-}$ mice treated with MEL-14 or IgG2a antibodies at day 5 p.i. with vaccNP.

Bar charts show means ± SEM. $^*p < 0.05$; $^{**}p < 0.005$.

effector $CD8^+$ T cell homing to this LN during an ongoing immune response to virus infection (Figure 5C). The enrichment of L-selectin-expressing activated $CD8^+$ T cells in ovaries of vaccinia-infected mice in the first 3 hr following intravenous administration demonstrates that L-selectin controls homing of activated $CD8^+$ T cells from the bloodstream into virus-infected tissues.

L-selectin-Dependent Homing of $CD8^+$ T Cells to Sites of Vaccinia Virus Infection Confers Protective Anti-virus Immunity

We hypothesized that, if L-selectin-expressing $CD8^+$ T cells home better to vaccinia-infected tissues, they may enhance virus clearance. Therefore, we tested the ability of F5 $CD8^+$ T cells to clear vaccNP from immunodeficient mice. First, the kinetics of virus clearance from naive B6 and $RAG^{-/-}$ mice were compared to determine the optimal time point to assess lymphocyte-dependent virus clearance. As shown in Figure 6A, virus titers in B6 and $RAG^{-/-}$ mice were not significantly different at 5 days p.i.; however, lymphocyte-dependent virus clearance was seen at day 8, when virus titers fell by 2 logs in B6, but not in $RAG^{-/-}$ mice. To determine whether L-selectin-expressing $CD8^+$ T cells promote virus clearance, $CD8^+$ T cells from naive F5/B6 or F5/LΔP mice were injected into $RAG^{-/-}$ mice. The following day, T-cell-injected and non-T-cell-injected $RAG^{-/-}$ mice were given vaccNP. All mice received interleukin (IL)-2 to enhance the proliferation and survival of transferred $CD8^+$ T cells in the absence of $CD4^+$ T cell help (Figure 6B). Virus titers were slightly reduced by transferring F5/B6 T cells. However, the virus load was markedly reduced by transferring F5 $CD8^+$ T cells expressing LΔP-selectin (Figure 6C). F5/LΔP $CD8^+$ T cells secreted similar levels of cytotoxic mediators such as IFN-γ, tumor necrosis factor α (TNF-α), and granzyme-B as wild-type cells (Figure 6D). Both cell types show similar levels of proliferation measured by incorporation of EdU (5-ethynyl-2′-deoxyuridine) dye into the DNA of proliferating F5 $CD8^+$ T cells (Figure 6E). As found in B6 mice, the homing of LΔP T cells to virus-infected ovaries was completely inhibited by MEL-14 (Figure 6F). Together, these results suggest that the enhanced virus clearance by $CD8^+$ T cells expressing LΔP-selectin is not due to increased numbers of polyfunctional T cells or increased rate of proliferation and, instead, correlates solely with increased L-selectin-dependent homing to virus-infected tissues.

L-selectin Expression Promotes Virus Clearance during Pulmonary Influenza Virus Infection

Although very informative for cellular studies of anti-virus immune responses, the vaccinia model is of limited clinical relevance. However, i.n. infection with influenza virus in mice can be considered closest to the natural route of human influenza infections. Having demonstrated that L-selectin regulates the homing of virus-specific $CD8^+$ T cells to influenza-infected lungs (Figure 4G), we determined whether L-selectin confers anti-virus immunity. $L\text{-sel}^{-/-}$, B6, and LΔP polyclonal mice were infected intranasally with two different strains of influenza A viruses, and lungs were harvested for virus titer 8 days p.i. Virus titers were significantly lower in wild-type mice than in L-selectin-deficient mice following infection with the pathogenic PR8 (H1N1) or the non-pathogenic H17 (H3N2) strains of influenza virus, demonstrating a role for L-selectin in the clearance of virus from the lungs (Figures 7A and 7B). Interestingly, PR8 virus titers were significantly lower in LΔP mice than in B6 mice. Since L-selectin expression is restricted to T lymphocytes in LΔP mice, this strongly supports a role for L-selectin on T lymphocytes in the control of virus replication. Interestingly, H17 titers were not

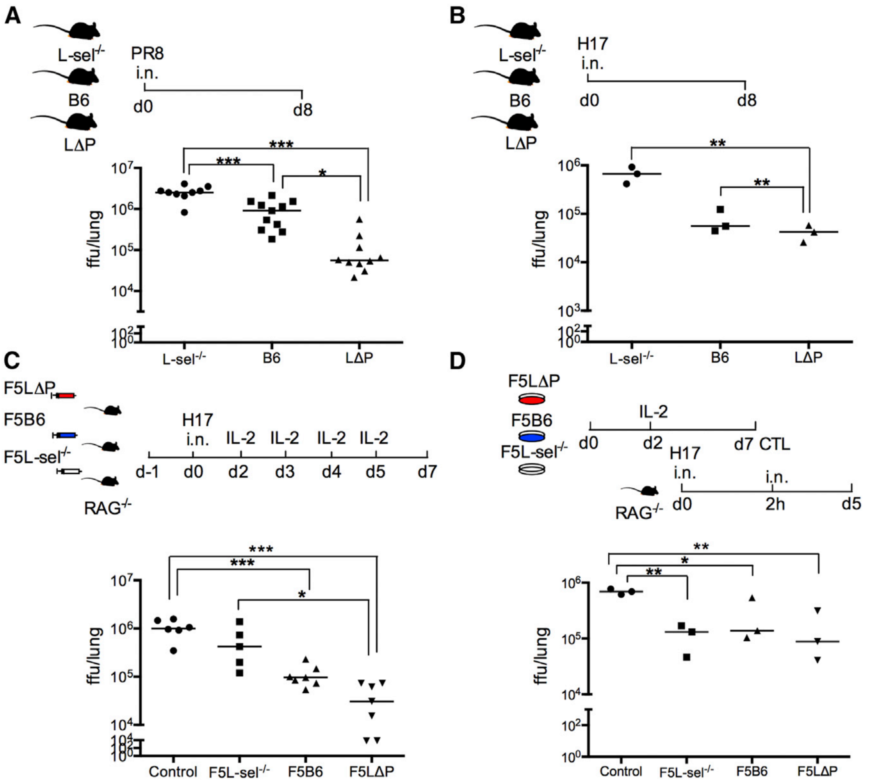

Figure 7. L-selectin Expression Promotes Protective Anti-virus Immunity during Primary Influenza Virus Infection

(A and B) Virus titers in the lungs of polyclonal L-sel$^{-/-}$, B6, and LΔP mice infected intranasally with influenza A virus strain (A) PR8 (H1N1) and (B) H17 (H3N2) at day (d) 8 p.i.

(C) Virus titers at day 7 p.i. in lungs of RAG$^{-/-}$ mice supplemented with naive F5L-sel$^{-/-}$, F5B6, or F5LΔP CD8$^+$ T cells intravenously or no T cells (control) and supplemented with IL-2 from days 2–5 p.i. with H17 influenza virus.

(D) Virus titers at day 5 p.i. in the lungs of RAG$^{-/-}$ mice infected with H17 influenza virus following installation of in-vitro-activated F5L-sel$^{-/-}$, F5B6, or F5LΔP CD8$^+$ T cells into the lungs. One-way ANOVA with post-Tukey's multiple comparison test.

*$p < 0.05$; **$p < 0.005$; ***$p < 0.0001$.

different in B6 and LΔP mice (Figures 7A and 7B), which may relate to the reduced pathogenicity of this virus. To determine whether L-selectin on effector CD8$^+$ T cells primed in draining LNs is sufficient to confer protective immunity to influenza virus, CD8$^+$ T cells from naive F5L-sel$^{-/-}$, F5B6, or F5LΔP mice were intravenously injected into RAG$^{-/-}$ mice, and 24 hr later, T-cell-injected and non-T-cell-injected RAG$^{-/-}$ mice were infected intranasally with H17 influenza virus and supplemented with IL-2 between days 2 and 5 p.i. (Figure 7C). Virus titers in RAG$^{-/-}$ mice supplemented with L-selectin-sufficient CD8$^+$ T cells were significantly reduced in comparison with non-T-cell-injected mice, and the higher levels of L-selectin on F5LΔP T cells conferred increased protection against influenza virus infection (Figure 7C). In contrast, virus titers in RAG$^{-/-}$ mice supplemented with L-selectin-deficient CD8$^+$ T cells were not statistically different from those in non-T-cell-injected mice. The increased homing of influenza-specific LΔP CD8$^+$ T cells to virus-infected tissues (Figure 2), rather than increased proliferation or polyfunctionality (Figure 6), suggests that the inability of L-selectin-deficient CD8$^+$ T cells to control virus replication is related to homing. To address this directly, in-vitro-generated cytotoxic T lymphocytes sufficient and deficient in L-selectin were instilled directly into the airways of influenza-infected mice. Cytotoxic T lymphocytes delivered intratracheally were able to control virus replication independently of L-selectin expression (Figure 7D). These data, therefore, demonstrate that, following priming in draining LNs and their release into the bloodstream, L-selectin is essential for the recruitment of protective, virus-specific CD8$^+$ T cells into infected tissues.

DISCUSSION

For the past 30 years, cellular immunologists have used L-selectin to determine the activation and differentiation status of T lymphocytes in different organs of the body, but the transient nature and role of L-selectin on activated T cells have been ignored. In this study, we show that L-selectin reversibly cycles between high and low expression during the differentiation of virus-specific effector CD8$^+$ T cells in vivo. Importantly, although L-selectin is downregulated within 24 hr at the site of priming in LNs, L-selectin re-expression starts before activated CD8$^+$ T cells exit LNs. Activated CD8$^+$ T cells entering the circulation have fully re-expressed L-selectin, and this re-expressed L-selectin regulates homing to virus-infected tissues. Furthermore, by promoting homing, L-selectin expression confers protective immunity to two evolutionarily distinct viruses during primary infection of different internal organs, demonstrating a critical role for this biological process in anti-virus CD8$^+$ T cell immunity.

We used two different viruses and routes of inoculation where CD8$^+$ T cell priming was restricted to a single LN and productive virus replication was restricted to a single tissue, lung or ovary. This allowed the kinetics and location of L-selectin downregulation and re-expression early after T cell activation to be determined in vivo. Activated T cells start to exit the LN 3 days following antigen administration (Fazilleau et al., 2009; Ford and Atkins, 1971; Mempel et al., 2004), and, as shown in this study, activated CD8$^+$ T cells are recruited into virus-infected tissue as early as day 3 p.i. when they have fully re-expressed L-selectin. We found no evidence that L-selectin regulates the recruitment or priming of CD8$^+$ T cells in mediastinal LN. Naive, L-selectin-deficient CD8$^+$ T cells migrated from the bloodstream into mediastinal LN as efficiently as L-selectin-sufficient CD8$^+$ T cells, a mechanism thought to be dependent upon $\alpha 4\beta 7$ integrin binding to MAdCAM-1 on high endothelial venules (HEV), as reported in other mucosal-tissue-associated LNs (Bargatze

et al., 1995; Hamann et al., 1994). The kinetics of CD8$^+$ T cell activation measured by CD69 expression and cell proliferation were similar in T cells deficient and sufficient in L-selectin. L-selectin did not regulate the subsequent differentiation of effector CD8$^+$ T cells either, since the fraction of proliferating cells and the expression of CD44 and other homing-associated molecules, intracellular cytokines, and granzymes were similar in L-selectin-sufficient and L-selectin-deficient T cells harvested at days 5 and 8 from virus-infected tissues, draining LN, and the spleen.

We used three different approaches to demonstrate that the expression of L-selectin on activated CD8$^+$ T cells promotes homing to virus-infected tissues. First, the enrichment of LΔP T cells expressing increased levels of L-selectin (Galkina et al., 2007) and the reduction in T cells deficient in L-selectin support the hypothesis that L-selectin regulates the recruitment of virus-specific T cells into infected organs.

Second, antibody blockade of L-selectin after T cells had been activated in mediastinal LN reduced recruitment of donor- and host-derived virus-specific effector CD8$^+$ T cells by 56%–85% during primary infection with vaccinia and influenza viruses. L-selectin blockade did not inhibit late priming of CD8$^+$ T cells (after 52 hr) following influenza infection, since the numbers of activated CD8$^+$ T cells in the mediastinal LN and spleen were not altered by MEL-14. There was also no evidence that L-selectin controlled late priming in ovary draining LNs, since the numbers of virus-specific CD8$^+$ T cells in the spleen were not altered by L-selectin blockade or L-selectin deficiency. Although there was a slight enrichment of F5/LΔP over F5/B6 CD8$^+$ T cells in ovary-draining LNs at day 5 p.i., this likely reflects increased L-selectin-dependent homing of F5/LΔP CD8$^+$ T cells that have retained cell-surface L-selectin following exit from mediastinal LN and entry into the bloodstream, rather than increased priming.

The third approach was to demonstrate the role of L-selectin in recruiting activated CD8$^+$ T cells from the bloodstream into virally infected tissues in a competitive short-term homing assay. Following injection in the bloodstream, L-selectin-sufficient and L-selectin-deficient activated CD8$^+$ T cells circulated equally, but after 3 hr, F5LΔP CD8$^+$ T cells were significantly enriched over F5L-sel$^{-/-}$ cells from 50% in the bloodstream to >85% in infected ovaries. Interestingly, in-vivo-generated effector T cells showed greater enrichment of L-selectin-expressing CD8$^+$ T cells than in-vitro-generated cells at 6.2- and 2.3-fold, respectively. This important finding suggests that the role of L-selectin in regulating effector T cell recruitment from the bloodstream into infected or inflamed tissues may be markedly underestimated, and that the role of other adhesion pathways may be overestimated, if in-vitro-generated T cells are used to study homing. The fact that L-selectin-deficient effector CD8$^+$ T cells are recruited into virus-infected tissues, albeit in low numbers, suggests that other molecules can substitute for L-selectin. Obvious candidates are the endothelial selectins. However, the recruitment of endogenously activated CD8$^+$ T cells to sites of virus-induced inflammation in the skin and internal organs is unchanged in E- and P-selectin double-knockout mice (Bartholdy et al., 2000). In contrast, E- and P-selectins have been reported to regulate effector CD8$^+$ T cell recruitment to hypersensitivity lesions in the skin (Hirata et al., 2002). Together, these results suggest that roles for E- and P-selectins, as opposed to L-selectin, in effector CD8$^+$ T cell recruitment may depend on the nature of the inflammatory stimulus and/or cytokine activation of local blood vessels. These results, combined with our own findings reported here, suggests that L-selectin is the dominant selectin pathway used by effector CD8$^+$ T cells in virus-infected tissues. The ligand is distinct from classical L-selectin ligands such as GlyCAM-1, CD34, podocalyxin, and MAdCAM-1, since MECA79 immunostaining of CD31-positive blood vessels was not detected in virus-infected lungs or ovaries.

Having demonstrated L-selectin-dependent homing of activated CD8$^+$ T cells to virus-infected tissues, we determined the impact of this pathway on virus elimination. Transfer of CD8$^+$ T cells to lymphocyte-deficient mice enhanced virus clearance, and the effect of T cells expressing high levels of L-selectin was greater (Figure 6C). Although L-selectin co-stimulates T cell proliferation and regulates the activity of cytotoxic T lymphocytes in vitro (Murakawa et al., 1992; Nishijima et al., 2005; Yang et al., 2011), the role of L-selectin in controlling vaccinia virus infection did not simply correlate with increased polyfunctionality or hyperproliferation. (Figure 6D) but instead correlates with increased homing. To determine whether L-selectin is required to recruit protective, virus-specific T cells to other internal organs, mice were intranasally infected with influenza A virus. Polyclonal LΔP mice, which selectively express shedding-resistant L-selectin on T cells (Galkina et al., 2003), were better able to resist influenza A challenge than wild-type mice, and L-selectin knockout mice were more susceptible to influenza A infection. These findings demonstrate the essential role of L-selectin in recruiting protective, virus-specific CD8$^+$ T cells to virus-infected mucosal as well as visceral organs and suggest that the effect of wild-type CD8$^+$ T cells is limited by the numbers that access the site of infection following T-cell-receptor (TCR)-induced downregulation of L-selectin. It is possible that loss of L-selectin expression by effector T cells evolved as a trade-off to limit immune-mediated pathology yet retain adaptive cell-mediated immune defense again pathogens during a primary infection.

It was originally hypothesized that L-selectin downregulation is required to redirect effector T cells away from LNs and allow migration to sites of inflammation, but our previous studies do not support this hypothesis (Richards et al., 2008), and, as shown in this study, L-selectin expression actually promotes homing of activated T cells to sites of virus infection. Our new data demonstrate that cyclical L-selectin expression forms a critical part of the arsenal of effector CD8$^+$ T cells by imparting the ability to home to virus infected tissues. It will be important to dissect the mechanisms that control cyclical expression of L-selectin in activated T cells, since the "window of opportunity" for L-selectin-dependent, protective immunity is time limited, and this may impact the persistence of immunological memory to viruses and vaccines. The results presented here indicate that maintaining expression of L-selectin on T cells boosts CD8$^+$ T cell immunity, which may have clinical relevance in adoptive T cell therapies for virus infections or for tumors.

EXPERIMENTAL PROCEDURES

Mice

The generation of L-selectin transgenic and knockout mice have been described previously (Arbonés et al., 1994; Galkina et al., 2003), and the genotypes of mice used in this study are listed in Table S1. C57BL/6 mice were purchased from Harlan Laboratories or Charles River Laboratories. C57BL/6 RAG2$^{-/-}$ mice were from the Francis Crick Institute. All other mice were bred in house. All experiments were conducted according to Cardiff University institutional guidelines and UK Home Office regulations.

Viruses

VaccNP (Cerundolo et al., 1997) was injected intraperitoneally (i.p.) at 2 × 10^6 plaque-forming units (pfu). Influenza A virus strains A/PuertoRico/8/34 (PR8, H1N1) and E61-13-H17 (H17, H3N2) were obtained from the Francis Crick Institute, and mice were infected intranasally with a split dose of 50 focus-forming units (ffu) in 50 μl sterile PBS under light anesthesia, as described previously (Lauder et al., 2013).

Virus Titers

For vaccNP, virus titers were determined using a plaque assay on 70%–80% confluent TK− cells as described previously (Jones et al., 2003). For PR8 and H17, a virus titration assay was used as previously described (Bachmann et al., 1999), except that serially diluted lung homogenates were incubated with 1.4 × 10^5 trypsinized Luc9.1 clone of Madin Darby canine kidney (MDCK) cells (Hossain et al., 2010). Each stained, influenza-infected MDCK cell was recorded immediately after staining as single ffu, and the total ffu per lung were calculated.

CD8$^+$ T Lymphocyte Isolation

Spleens and/or LNs were harvested from adult mice and mashed through a 70-μm cell strainer (BD Pharmingen). Red blood cells were lysed using red blood cell lysis buffer (BioLegend), and lymphocytes were washed with ice-cold PBS supplemented with 2% fetal calf serum (FCS). CD8$^+$ T lymphocytes were isolated using the MACS (magnetic-activated cell sorting) column negative selection technique (Miltenyi Biotec) according to the manufacturer's instructions. After isolation, cells were stained with anti-CD8α antibody to check the purity (>98%).

Adoptive Transfer of CD8$^+$ T Cells

2 × 10^6 naive purified CD8$^+$ T lymphocytes from F5/LΔP, F5/B6, F5B6, or F5L-sel$^{-/-}$ mice (all CD90.2$^+$) in 200 μl saline were injected intravenously (i.v.) into the tail vein of groups of three or four age- and sex-matched CD90.2$^+$ B6, RAG1$^{-/-}$, or RAG2$^{-/-}$ mice and left for 24 hr to allow the transferred lymphocytes to circulate and equilibrate in the recipient mice. One group of mice was injected with saline and used as a no-T-cell control. For the CD8$^+$ T cell priming experiment, naive CD8$^+$ T cells were purified from spleens and then washed with PBS and resuspended in 10 μM carboxyfluorescein succinimidyl ester (CFSE; Life Technologies/Invitrogen), 2 μM PKH-26, or 2 μM Cell Vue Claret (Sigma-Aldrich) and incubated in the dark for 10 min at room temperature. Equal volumes of FCS were added, and the cells then washed twice with PBS, re-suspended in saline, and injected into CD90.1$^+$ B6 mice.

Flow Cytometry

Mice were euthanized, blood was collected by cardiac puncture, and tissues were excised. Mediastinal, axillary, inguinal, and ovary-draining LNs, ovaries, lungs, and spleens were harvested in PBS and mashed through a 70-μm cell strainer. Lungs were digested in 1 mg/ml collagenase D (Roche) and 10 μg/ml DNase I (USB) for 45 min at 37°C prior to mashing. Red cells were lysed in blood, spleen, and lung preparations. Isolated cells were washed with PBS and 2% FCS and stained with fixable viability dye (Amcyan; Invitrogen) as per manufacturer's protocol, and cells were stained for tetramer and cell-surface markers with a combination of antibodies listed in the Supplemental Experimental Procedures.

Short-Term Homing Assays

F5L-sel$^{-/-}$ and F5/LΔP CD8+ T cells were activated either in vitro or in vivo for 7 and 5 days, respectively. Cells were mixed 1:1 and transferred i.v. into vaccNP-infected B6 mice at 3 days p.i. After 3 hr, organs were harvested, and donor cells were analyzed as described in the Supplemental Experimental Procedures.

Statistical Analysis and Figures

Linear results are presented as means ± SEM from three to six mice per group. Viral titers in individual mice and median values pooled from two independent experiments (3–12 mice per group) are shown on a log scale. Figures were drawn, and statistical analyses were performed using GraphPad Prism software, Mac version 5.1, and statistical significance between groups was determined using an unpaired Student's t test, unless otherwise stated.

SUPPLEMENTAL INFORMATION

Supplemental Information includes Supplemental Experimental Procedures, two figures, and one table and can be found with this article online at http://dx.doi.org/10.1016/j.celrep.2015.12.090.

AUTHOR CONTRIBUTIONS

R.N.M. and A.A. conceived the study, and R.N.M. performed and analyzed all the experiments, with assistance from all authors. A.A. supervised all aspects of the study, with critical input from H.A.W. and I.R.H. R.N.M. and A.A. wrote the manuscript, with contributions from all authors.

ACKNOWLEDGMENTS

We thank Awen Gallimore and Shahram Salek-Ardakani for advice on virus infections; Sarah Lauder, Kathryn Smart, and Sophie-Marie Forstner for influenza virus titers; Kristin Ladell for advice on flow cytometry; Graham Preece and Geoffrey Butcher for antibodies; Ruben Donis and the Centers for Disease Control and Prevention for Luc9.1 MDCK cells; and Martina di Nunzio for suggesting using them. We acknowledge the NIH Tetramer Core Facility (contract HHSN272201300006C) for provision of H-2D(b) Influenza A NP 366-374 ASNENMETM monomer. We also thank Thomas Tedder for L-selectin knockout mice, as well as Sophie Wehenkel, Garry Dolton, Simon Jones, Edward Wang, and David Price for extremely helpful comments on the manuscript. Finally, we thank Biological Services at Cardiff University for mouse care. This work was supported by the Ministry of Higher Education and Scientific Research of the Kurdistan Regional Government, Iraq; and project grants from the Wellcome Trust (094511/Z/10/Z) and the Medical Research Council UK (MR/L008742/1).

Received: September 4, 2015
Revised: December 1, 2015
Accepted: December 18, 2015
Published: January 21, 2016

REFERENCES

Arbonés, M.L., Ord, D.C., Ley, K., Ratech, H., Maynard-Curry, C., Otten, G., Capon, D.J., and Tedder, T.F. (1994). Lymphocyte homing and leukocyte rolling and migration are impaired in L-selectin-deficient mice. Immunity *1*, 247–260.

Austrup, F., Vestweber, D., Borges, E., Löhning, M., Bräuer, R., Herz, U., Renz, H., Hallmann, R., Scheffold, A., Radbruch, A., and Hamann, A. (1997). P- and E-selectin mediate recruitment of T-helper-1 but not T-helper-2 cells into inflammed tissues. Nature *385*, 81–83.

Bachmann, M.F., Ecabert, B., and Kopf, M. (1999). Influenza virus: a novel method to assess viral and neutralizing antibody titers in vitro. J. Immunol. Methods *225*, 105–111.

Bargatze, R.F., Jutila, M.A., and Butcher, E.C. (1995). Distinct roles of L-selectin and integrins alpha 4 beta 7 and LFA-1 in lymphocyte homing to Peyer's patch-HEV in situ: the multistep model confirmed and refined. Immunity *3*, 99–108.

Bartholdy, C., Marker, O., and Thomsen, A.R. (2000). Migration of activated CD8(+) T lymphocytes to sites of viral infection does not require endothelial selectins. Blood *95*, 1362–1369.

Cerundolo, V., Benham, A., Braud, V., Mukherjee, S., Gould, K., Macino, B., Neefjes, J., and Townsend, A. (1997). The proteasome-specific inhibitor lactacystin blocks presentation of cytotoxic T lymphocyte epitopes in human and murine cells. Eur. J. Immunol. *27*, 336–341.

Chao, C.C., Jensen, R., and Dailey, M.O. (1997). Mechanisms of L-selectin regulation by activated T cells. J. Immunol. *159*, 1686–1694.

DeGrendele, H.C., Estess, P., and Siegelman, M.H. (1997). Requirement for CD44 in activated T cell extravasation into an inflammatory site. Science *278*, 672–675.

Fazilleau, N., McHeyzer-Williams, L.J., Rosen, H., and McHeyzer-Williams, M.G. (2009). The function of follicular helper T cells is regulated by the strength of T cell antigen receptor binding. Nat. Immunol. *10*, 375–384.

Ford, W.L., and Atkins, R.C. (1971). Specific unresponsiveness of recirculating lymphocytes ater exposure to histocompatibility antigen in F 1 hybrid rats. Nat. New Biol. *234*, 178–180.

Fung-Leung, W.P., Kündig, T.M., Zinkernagel, R.M., and Mak, T.W. (1991). Immune response against lymphocytic choriomeningitis virus infection in mice without CD8 expression. J. Exp. Med. *174*, 1425–1429.

Galkina, E., Tanousis, K., Preece, G., Tolaini, M., Kioussis, D., Florey, O., Haskard, D.O., Tedder, T.F., and Ager, A. (2003). L-selectin shedding does not regulate constitutive T cell trafficking but controls the migration pathways of antigen-activated T lymphocytes. J. Exp. Med. *198*, 1323–1335.

Galkina, E., Florey, O., Zarbock, A., Smith, B.R., Preece, G., Lawrence, M.B., Haskard, D.O., and Ager, A. (2007). T lymphocyte rolling and recruitment into peripheral lymph nodes is regulated by a saturable density of L-selectin (CD62L). Eur. J. Immunol. *37*, 1243–1253.

Gallatin, W.M., Weissman, I.L., and Butcher, E.C. (1983). A cell-surface molecule involved in organ-specific homing of lymphocytes. Nature *304*, 30–34.

Goulding, J., Abboud, G., Tahiliani, V., Desai, P., Hutchinson, T.E., and Salek-Ardakani, S. (2014). CD8 T cells use IFN-γ to protect against the lethal effects of a respiratory poxvirus infection. J. Immunol. *192*, 5415–5425.

Hamann, A., Andrew, D.P., Jablonski-Westrich, D., Holzmann, B., and Butcher, E.C. (1994). Role of alpha 4-integrins in lymphocyte homing to mucosal tissues in vivo. J. Immunol. *152*, 3282–3293.

Hickman, H.D., Reynoso, G.V., Ngudiankama, B.F., Cush, S.S., Gibbs, J., Bennink, J.R., and Yewdell, J.W. (2015). CXCR3 chemokine receptor enables local CD8(+) T cell migration for the destruction of virus-infected cells. Immunity *42*, 524–537.

Hirata, T., Furie, B.C., and Furie, B. (2002). P-, E-, and L-selectin mediate migration of activated CD8+ T lymphocytes into inflamed skin. J. Immunol. *169*, 4307–4313.

Hossain, M.J., Perez, S., Guo, Z., Chen, L.M., and Donis, R.O. (2010). Establishment and characterization of a Madin-Darby canine kidney reporter cell line for influenza A virus assays. J. Clin. Microbiol. *48*, 2515–2523.

Jones, E., Price, D.A., Dahm-Vicker, M., Cerundolo, V., Klenerman, P., and Gallimore, A. (2003). The influence of macrophage inflammatory protein-1alpha on protective immunity mediated by antiviral cytotoxic T cells. Immunology *109*, 68–75.

Kaech, S.M., Hemby, S., Kersh, E., and Ahmed, R. (2002). Molecular and functional profiling of memory CD8 T cell differentiation. Cell *111*, 837–851.

Lauder, S.N., Jones, E., Smart, K., Bloom, A., Williams, A.S., Hindley, J.P., Ondondo, B., Taylor, P.R., Clement, M., Fielding, C., et al. (2013). Interleukin-6 limits influenza-induced inflammation and protects against fatal lung pathology. Eur. J. Immunol. *43*, 2613–2625.

Lefrançois, L., and Marzo, A.L. (2006). The descent of memory T-cell subsets. Nat. Rev. Immunol. *6*, 618–623.

Liu, L., Fuhlbrigge, R.C., Karibian, K., Tian, T., and Kupper, T.S. (2006). Dynamic programming of CD8+ T cell trafficking after live viral immunization. Immunity *25*, 511–520.

Masopust, D., Vezys, V., Usherwood, E.J., Cauley, L.S., Olson, S., Marzo, A.L., Ward, R.L., Woodland, D.L., and Lefrançois, L. (2004). Activated primary and memory CD8 T cells migrate to nonlymphoid tissues regardless of site of activation or tissue of origin. J. Immunol. *172*, 4875–4882.

Mempel, T.R., Henrickson, S.E., and Von Andrian, U.H. (2004). T-cell priming by dendritic cells in lymph nodes occurs in three distinct phases. Nature *427*, 154–159.

Mobley, J.L., and Dailey, M.O. (1992). Regulation of adhesion molecule expression by CD8 T cells in vivo. I. Differential regulation of gp90MEL-14 (LECAM-1), Pgp-1, LFA-1, and VLA-4 alpha during the differentiation of cytotoxic T lymphocytes induced by allografts. J. Immunol. *148*, 2348–2356.

Mora, J.R., and von Andrian, U.H. (2006). T-cell homing specificity and plasticity: new concepts and future challenges. Trends Immunol. *27*, 235–243.

Murakawa, Y., Minami, Y., Strober, W., and James, S.P. (1992). Association of human lymph node homing receptor (Leu 8) with the TCR/CD3 complex. J. Immunol. *148*, 1771–1776.

Nishijima, K., Ando, M., Sano, S., Hayashi-Ozawa, A., Kinoshita, Y., and Iijima, S. (2005). Costimulation of T-cell proliferation by anti-L-selectin antibody is associated with the reduction of a cdk inhibitor p27. Immunology *116*, 347–353.

Oehen, S., and Brduscha-Riem, K. (1998). Differentiation of naive CTL to effector and memory CTL: correlation of effector function with phenotype and cell division. J. Immunol. *161*, 5338–5346.

Richards, H., Longhi, M.P., Wright, K., Gallimore, A., and Ager, A. (2008). CD62L (L-selectin) down-regulation does not affect memory T cell distribution but failure to shed compromises anti-viral immunity. J. Immunol. *180*, 198–206.

Siegelman, M.H., Stanescu, D., and Estess, P. (2000). The CD44-initiated pathway of T-cell extravasation uses VLA-4 but not LFA-1 for firm adhesion. J. Clin. Invest. *105*, 683–691.

Tang, M.L., Hale, L.P., Steeber, D.A., and Tedder, T.F. (1997). L-selectin is involved in lymphocyte migration to sites of inflammation in the skin: delayed rejection of allografts in L-selectin-deficient mice. J. Immunol. *158*, 5191–5199.

Tedder, T.F., Steeber, D.A., and Pizcueta, P. (1995). L-selectin-deficient mice have impaired leukocyte recruitment into inflammatory sites. J. Exp. Med. *181*, 2259–2264.

Townsend, A.R., Rothbard, J., Gotch, F.M., Bahadur, G., Wraith, D., and McMichael, A.J. (1986). The epitopes of influenza nucleoprotein recognized by cytotoxic T lymphocytes can be defined with short synthetic peptides. Cell *44*, 959–968.

Yang, S., Liu, F., Wang, Q.J., Rosenberg, S.A., and Morgan, R.A. (2011). The shedding of CD62L (L-selectin) regulates the acquisition of lytic activity in human tumor reactive T lymphocytes. PLoS ONE *6*, e22560.

Zhang, N., and Bevan, M.J. (2011). CD8(+) T cells: foot soldiers of the immune system. Immunity *35*, 161–168.

Zhao, Y., Adams, Y.F., and Croft, M. (2011). Preferential replication of vaccinia virus in the ovaries is independent of immune regulation through IL-10 and TGF-β. Viral Immunol. *24*, 387–396.

Zinkernagel, R.M., and Doherty, P.C. (1979). MHC-restricted cytotoxic T cells: studies on the biological role of polymorphic major transplantation antigens determining T-cell restriction-specificity, function, and responsiveness. Adv. Immunol. *27*, 51–177.

Cell Reports

Supplemental Information

L-selectin Is Essential for Delivery of Activated CD8$^+$ T Cells to Virus-Infected Organs for Protective Immunity

Rebar N. Mohammed, H. Angharad Watson, Miriam Vigar, Julia Ohme, Amanda Thomson, Ian R. Humphreys, and Ann Ager

Supplemental Information

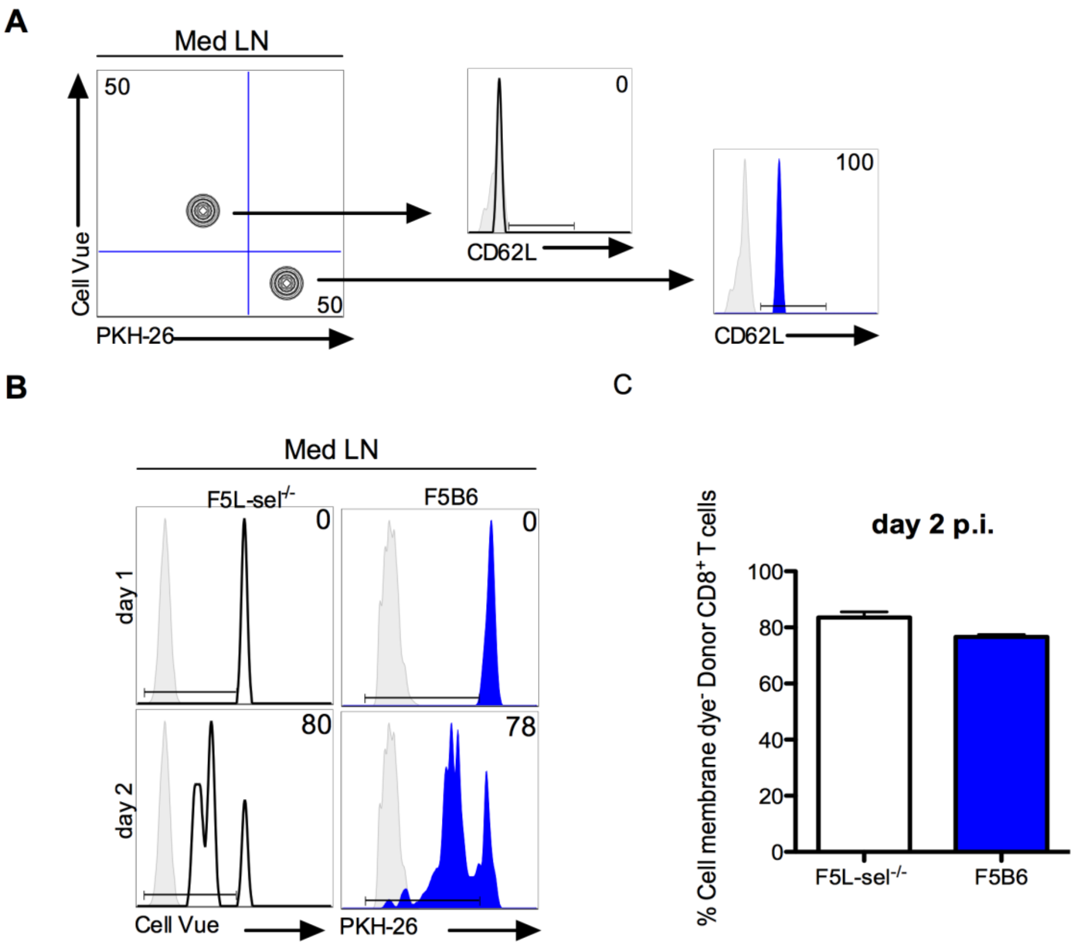

Figure S1, Related to Figure 2: L-selectin expression does not affect localization or priming of $CD8^+$ T cells in the mediastinal LN. Naïve F5L-$sel^{-/-}$ and F5B6 $CD8^+$ T cells were purified and stained with 2µM Cell Vue Claret and PKH-26 respectively. Cells were mixed 1:1 and total of 4 x 10^6 were transferred i.v. into CD90.1 B6 mice. Mice were infected with 2 x 10^6 pfu vaccNP or left un-infected. **(A)** Representative plot shows the percentage of F5B6 (PKH-26^+) and F5L-$sel^{-/-}$ (Cell Vue^+) $CD8^+$ T cells in the Mediastinal LN in un-infected mice 24h post transfer; the donor cells were confirmed by CD62L expression. **(B)** Representative histograms show the percentage cell tracker dye dilution at days 1 and 2 p.i. compared to the unstained cell, grey histograms. **(C)** Bar chart shows the mean + SEM dye dilution (n=3).

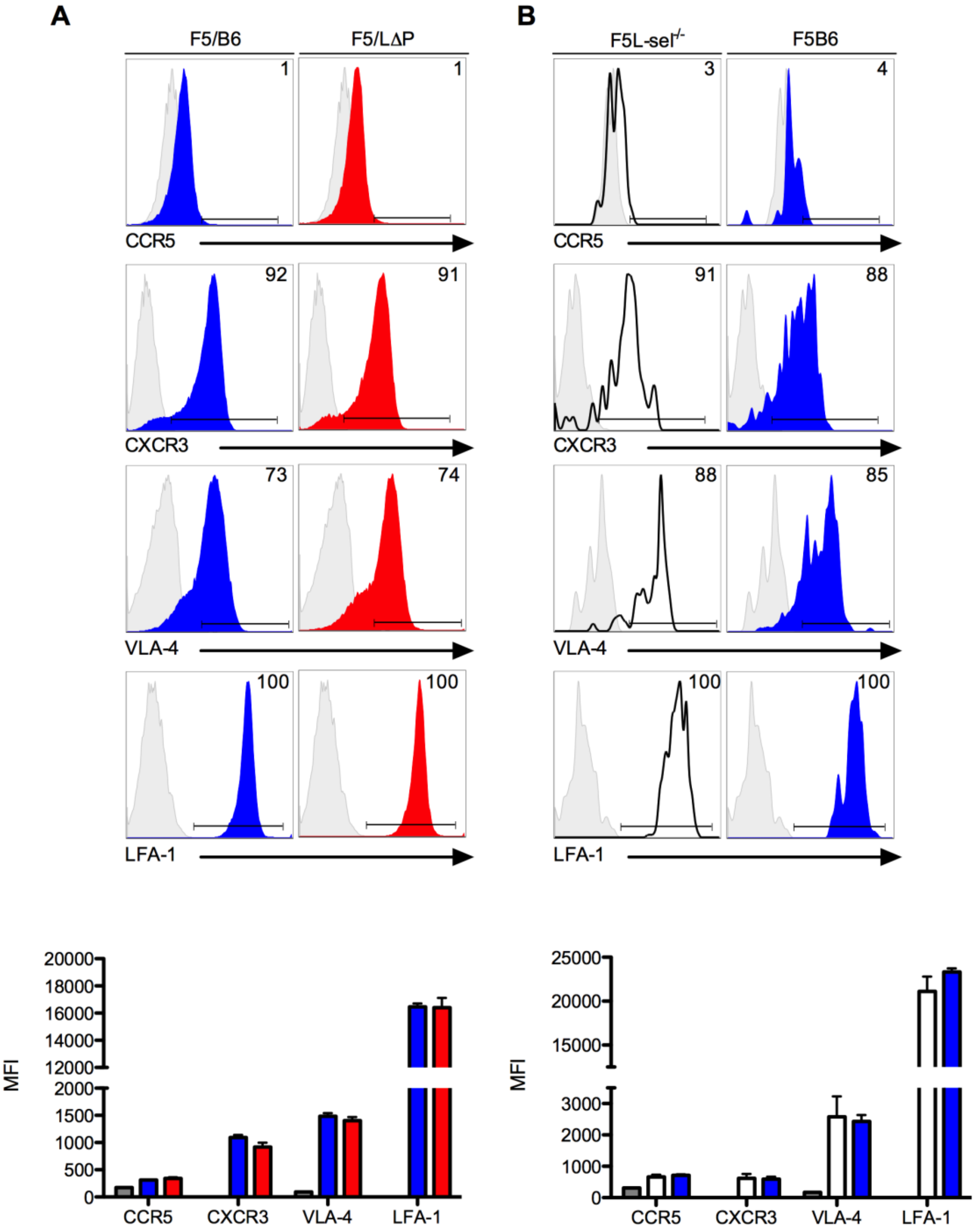

A
F5/B6
F5/LΔP
CCR5
CXCR3
VLA-4
LFA-1
1
1
92
91
73
74
100
100
B
F5L-sel-/-
F5B6
3
4
91
88
88
85
100
100
MFI
20000
18000
16000
14000
12000
2000
1500
1000
500
0
25000
20000
15000
3000
2000
1000
0
CCR5
CXCR3
VLA-4
LFA-1

Figure S2, Related Figure 3: L-selectin does not affect expression of inflammation associated homing molecules on virus specific CD8$^+$ T cells. (A) F5/B6 (blue) and F5/LΔP (red) or **(B)** F5L-sel$^{-/-}$ (open) and F5B6 (blue) CD8$^+$ T cells were transferred into naïve B6 mice and 24 hours later mice given vaccNP i.p. Spleens were collected 5 day p.i. and donor cells were identified as in Fig. 2. Representative histograms show percentage positive CCR5, CXCR3, VLA-4 (CD49d) and LFA-1 (CD11a) by donor CD8$^+$ T cells. Bar charts show mean $\pm$ SEM MFI (n=4).

Table S1, Related to Experimental Procedures: Mice

Mice	F5 genotype	Endogenous L-selectin genotype	Transgenic LΔP genotype	CD90 genotype	Fold increase L-selectin expression over wildtype
F5/B6	+/-	+/+	-	CD90.2	1.0
F5/LΔP	+/-	-/-	+/-	CD90.2	1.5[a]
F5/ CD90.1B6	+/-	+/+		CD90.1/CD90.2	1.0
F5B6	+/+	+/+	-	CD90.2	1.0
F5LΔP	+/+	-/-	+/+	CD90.2	3.0[a]
F5L-sel$^{-/-}$	+/+	-/-	-	CD90.2	No expression
LΔP	-/-	-/-	+/+	CD90.2	3.0[a]
L-sel$^{-/-}$	-/-	-/-	-/-	CD90.2	No expression
C57BL/6 (B6)	-/-	+/+	-/-	CD90.2	1.0

[a] Data from (Galkina et al., 2007)

Supplemental Experimental Procedures

Flow Cytometry

Single cell isolate stained with PE-conjugated H2-D^b NP68-tetramer (Influenza H17 derived nucleoprotein 366-374 ASNENMDAM) or H-2D^b NP34-tetramer (Influenza A-PR8–34 nucleoprotein 366-374 ASNENMETM) at 37°C, followed by surface stains at 4°C with FITC-anti Vβ11 (clone: KT-11, Biolegend), PerCPCy5.5-anti CD8 (53-6.7, Biolegend), PECy7-anti CD62L (MEL-14, Biolegend), APC-anti CD69 (H1.2F3, Biolegend), APCCy7-anti CD44 (IM7, Biolegend), Pacific Blue-anti CD90.2/Thy1.2 (53-2.1, Biolegend), AlexaFluor 488-anti CD90.2 (30-H12, Biolegend) and intracellular stains with PerCPCy5.5-anti IFN-γ (XMG1.2, Biolegend), PE-Cy7-anti TNF-α (TN3-19, eBioscience), eFluor-450-anti IL-2 (JES6-5H4, eBiosceince) FITC anti-granzymeB (GB-11, Biolegend), Biotinylated-anti CCR5 (HM-CCR5 7A4, eBioscience), APC-anti CXCR3 (CXCR3-173, eBioscience), PE-anti CD49d (PS/2, Southern Biotechnology), PECy7-anti CD11a (M17/4, Biolegend). Fluorescence minus one (FMO) was used for non-specific, background staining. Cells were fixed and analyzed on a BD FACS Canto II flow cytometer and counted using Cytocount beads (DAKO). Figures were prepared using FlowJo software.

Short term homing assays

For *in vitro* activated CD8+ T cell experiment, F5LΔP and F5L-$sel^{-/-}$ $CD8^+$ T cells were purified and then re-suspended in complete medium (DMEM supplemented with 10% FCS, penicillin-streptomycin, L-glutamine, non-essential amino-acids and 2-β mercaptoethanol) and plated at 2 x 10^6 cells/well in 24-well plates (Nunclon). Splenocytes from F5B6 mice were pulsed with 5 µg/ml NP68 peptide (Peptide Synthetics) for 1 hour at 37°C, irradiated at 3000 centrigrays, 6 x10^6 splenocytes added per well and plates incubated at 37°C in 5% CO_2. Fresh complete medium supplemented with 360 IU/ml hrIL-2 was added after 2 days. Cells were harvested at day 7, mixed 1:1 and a total of 5.58 x 10^6 cells (2.76 x 10^6 F5LΔP and 2.82 x 10^6 F5L-$sel^{-/-}$ determined using Cytocount beads) injected i.v. into 4 vaccNP infected $CD90.1^+$ B6 mice. After 3 hours, ovaries, ovdLNs, axillary lymph nodes, inguinal lymph nodes, mediastinal lymph node, spleen, peripheral blood were harvested, stained for CD8, CD90.2 and L-selectin and analyzed by flow cytometry for numbers and L-selectin expression on donor $CD8^+$, $CD90.2^+$ cells. For in vivo activated CD8+ T cells, F5LΔP and F5L-$sel^{-/-}$ mice were infected with 2x10^6 vaccNP i.p., At day 5 post infection, mice were euthanized and activated $CD8^+$ T cells were isolated from spleen, mixed 1.16:0.84 and a total of 13.68 x 10^6 cells (5.8 x 10^6 F5LΔP and 7.87 x 10^6 F5L-$sel^{-/-}$) injected into 4 vaccNP infected $CD90.1^+$ B6 mice.

L-selectin inhibition and T cell proliferation in virus-infected mice

To inhibit L-selectin function, 250 µg of monoclonal antibody to mouse L-selectin (MEL-14) or rat IgG2a isotype control antibodies MAC193 (Price et al., 1997) or MAC 219 (Forsyth et al., 2000) were injected i.v.and/or i.p. either once or daily commencing 52 h post infection. The thymidine analogue 5-ethynyl-2'deoxyuridine (EdU; Life Technology,) was re-suspended in saline and 1mg injected i.p. 24 hour prior to tissue harvest.

rIL-2

Human recombinant interleukin-2 (hrIL-2) (Proleukin, Novartis Pharma,) was diluted in saline and $RAG1^{-/-}$ mice injected daily with 150,000 IU hrIL-2.

Supplemental References

Forsyth, I.A., Hutchings, A., and Butcher, G.W. (2000). A panel of monoclonal antibodies to ovine placental lactogen. J Endocrinol *165*, 435-442.

Galkina, E., Florey, O., Zarbock, A., Smith, B.R., Preece, G., Lawrence, M.B., Haskard, D.O., and Ager, A. (2007). T lymphocyte rolling and recruitment into peripheral lymph nodes is regulated by a saturable density of L-selectin (CD62L). Eur J Immunol *37*, 1243-1253.

Price, A.A., Cumberbatch, M., Kimber, I., and Ager, A. (1997). Alpha 6 integrins are required for Langerhans cell migration from the epidermis. J. Exp. Med. *186*, 1725-1735.
